# Analyzing the Modification of the *Shewanella oneidensis* MR-1 Flagellar Filament

**DOI:** 10.1371/journal.pone.0073444

**Published:** 2013-09-06

**Authors:** Sebastian Bubendorfer, Mayumi Ishihara, Kim Dohlich, Christian Heiss, Jan Vogel, Federico Sastre, Maria Panico, Paul Hitchen, Anne Dell, Parastoo Azadi, Kai M. Thormann

**Affiliations:** 1 Department of Ecophysiology, Max Planck Institute for Terrestrial Microbiology, Marburg, Germany; 2 Complex Carbohydrate Research Center, University of Georgia, Athens, Georgia, United States of America; 3 Division of Molecular Biosciences, Imperial College London, London, United Kingdom; 4 Department of Cellular Microbiology, Max Planck Institute for Infection Biology, Berlin, Germany; 5 Departamento de Ingeniería Química y Bioprocesos, Pontificia Universidad Católica de Chile, Santiago, Chile; Research Center Borstel, Germany

## Abstract

The unsheathed flagellar filament of *Shewanella oneidensis* MR-1 is composed of two highly homologous flagellins, FlaA, and the major structural unit, FlaB. We identified a gene cluster, SO_3261-SO_3265 (now *sfmABCDE*), that is required for the formation of a fully functional filament and for motility. The predicted function of the corresponding gene products strongly indicated a role in flagellin modification. Accordingly, loss of *sfmABCDE* results in a significant mass shift of both FlaA and FlaB. Mass spectroscopy analysis and single residue substitutions identified five serine residues in both flagellins that are modified via O-linkage. Modeling of the flagellin structures strongly suggests that at least four of the modified residues are exposed to the filament’s surface. However, none of the five serine residues solely is crucial for function and assembly. Structural analysis of the flagellin modification revealed that it likely contains a nonulosonic acid (274 Da) linked to each glycosylated serine. The putative nonulosonic acid is further substituted with a 236 Da moiety which can carry additional methyl groups (250 Da, 264 Da). In addition, at least 5 lysine residues in FlaB and one in FlaA were found to be methylated. Based on homology comparisons we suggest that *smfABCDE* is required for species-specific flagellin modification in *S. oneidensis* MR-1.

## Introduction

For many bacteria, the capability to actively move towards more favorable conditions provides an important advantage for successful propagation and survival [1], [2]. Numerous bacterial species are motile by means of flagella, long proteinaceous filaments extending from the cell body that are rotated at the filament’s base by a membrane-embedded motor. Flagella-mediated motility provides a very effective means of bacterial locomotion through liquid environments as well as across surfaces [3]. In addition, flagellar systems have been demonstrated to serve as environmental sensors [4], e.g. in determining the environment’s wetness or viscosity [5], [6]. The flagellar system consists of three major parts: the flagellar filament, the basal body, which includes the flagellar motor and the export machinery, and the hook which serves as a joint to connect basal body and filament [7]. The flagellar filament is composed of numerous flagellin proteins and, as the whole flagellar structure, is built from inside out: The flagellin subunits are exported by a type III secretion system to be assembled at the distal end of the filament [8], [9]. While the filament of many bacterial species consists of multiple copies of a single flagellin protein, a number of species, such as *Aeromonas hydrophila*, *Bdellovibrio bacteriovorus*, *Campylobacter* sp., *Caulobacter crescentus*, or *Vibrio* sp. possess flagella which are composed of several different flagellins [10]–[14].

The flagellar filaments of some species, such as the polar flagellum of *Vibrio* sp., are sheathed in a membrane-like structure [10], [15]. Another common modification of the flagellar filament is the glycosylation of the hydroxyl groups of serine or threonine residues within the flagellin subunits, referred to as O-(linked) glycosylation. A growing number of studies on a wide range of bacterial species suggests that this means of modification occurs rather frequently among species that thrive within the intestinal gut but also in other environments. The analysis of some flagellin-associated glycans revealed a remarkable variety of structures between the different bacterial species [16], [17]. Flagellin glycosylation is thought to impact flagellar stability and filament assembly and might play a role in host-microbe interaction, antigenic diversity and immune evasion [18]. The best studied bacterial O-linked glycosylation system with respect to flagellin modification is that of *Campylobacter* species. For this organism, pseudaminic acid (Pse) is among the predominant O-linked glycans, and the linkage occurs prior to the transport of the flagellin and assembly of the filament. The flagellin of *Helicobacter pylori* has been demonstrated to be similarly decorated with Pse [17], [19]–[21], and a recent study on *A. hydrophila* also strongly suggests a role of Pse in flagellin glycosylation of both polar and lateral flagellar systems [22].

Species of the genus *Shewanella* belong to the group of facultatively anaerobic gammaproteobacteria. Generally, all members of this genus exhibit a pronounced respiratory flexibility that allows them to use a wide range of alternative electron acceptors when growing under anaerobic conditions, among them numerous metal ions, including radionuclides, and halogenated compounds [23]–[25]. Thus, *Shewanella* species are thought to be suitable organisms for applications in bioremediation of contaminated soils or production of microbial fuel cells [26]–[28]. Some species of this highly diverse group have also emerged as opportunistic pathogens [29]. All members of the genus *Shewanella* are motile by at least one flagellar system, forming a single sodium ion-driven polar flagellum. For *S. oneidensis* MR-1, flagella-mediated motility allows a directed movement towards redox-active surfaces [30]. In addition, the functional flagellar system is required for normal biofilm formation at solid-liquid and liquid-air interfaces [31], [32]. A recent study on the flagellar assembly of *S. oneidensis* has demonstrated the presence and differential regulation of two flagellins, FlaA and FlaB [33]. The same study provides convincing evidence that both FlaA and FlaB are modified by glycosylation. Based on genetic approaches, the authors identified a PseB homolog in *Shewanella* and demonstrate a role of the protein in flagellin modification. However, the exact mechanism of flagellin modification in *Shewanella* and the factors involved therein are still mostly unknown. In this study, we identified a novel gene cluster whose gene products are involved in flagellin modification. We show that the major subunit FlaB is likely modified at five different surface-exposed serine residues, and we further characterized the modification.

## Results

### Identification of a Novel Gene Cluster Involved in Flagella-mediated Motility

**Figure 1:**
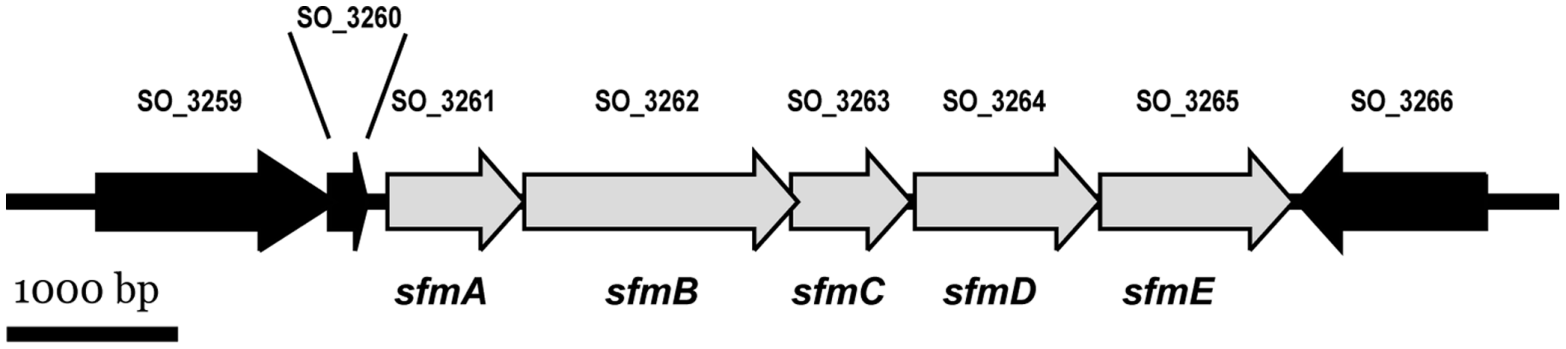
Genetic organization of the SO_3261– SO_3265 (*sfmABCDE*) gene cluster in *S. oneidensis* MR-1. The genes forming the operon are displayed in grey, the flanking genes which are most likely not part of the operon are shown in black.

During the course of a previous study, a transposon mutagenesis was conducted on *S. oneidensis* MR-1 using a Tn*5*-based transposon system. The resulting mutants were screened for alterations in their ability to form biofilms in a static microtitre plate assay [31]. Three transposon integrations mapped to neighboring genes SO_3263-SO_3265. All strains were severely affected in their ability to form biofilms under static conditions, and a secondary physiological screening revealed that this was probably due to a drastic decrease in flagella-mediated swimming (data not shown). All three genes belong to a putative five-gene operon also comprising SO_3261 and SO_3262 (Fig. 1). A FliA(δ^28^) consensus sequence (TAAAG-N_14_-GCCGTTAA) is predicted to be located 57 bp upstream of SO_3261 [34]. Accordingly, RT-PCR indicates that the gene SO_3259 (encoding a putative motility accessory factor, MAF) which located upstream is not co-transcribed with SO_3261-3265 (Fig. S1). Similarly, also the small open reading frame SO_3260 (encoding a hypothetical protein) is, most likely, not part of the operon. The gene is 873 bp in length, encoding a protein of 290 amino acids with a predicted molecular mass of 32.7 kDa. SO_3261 has a highly conserved N-acetylneuraminic acid synthase (NeuB) domain and, thus, is likely involved in biosynthesis of a polysaccharide. SO_3262 (*ilvB*) is 1746 bp in length, the deduced protein of 581 amino acids has a predicted molecular mass of 63.0 kDa. The protein has a thiamine pyrophosphate (TPP)-binding domain and has been annotated as TPP-dependent enzyme involved in flagellin modification. The third gene in the operon, SO_3263, has a length of 768 bp and encodes a protein of 255 amino acids, which is predicted to be a 3-oxoacyl-(acyl-carrier-protein) reductase. The fourth gene in the operon, SO_3264, 1188 bp, is predicted to encode a protein of 395 amino acids (44.7 kDa) with a putative function as an S-adenosylmethionine (SAM)-dependent methyltransferase. Finally, the last gene in the operon, SO_3265 (1239 bp; 412 amino acids; 44.0 kDa) contains an ARP-grasp_4 domain, suggesting a carboxylate-amine ligase or peptide modification activity [35]. All five proteins are predicted to localize to the cytoplasm.

### SO_3261- SO_3264 are Required for Normal Flagella-mediated Motility

**Figure 2:**
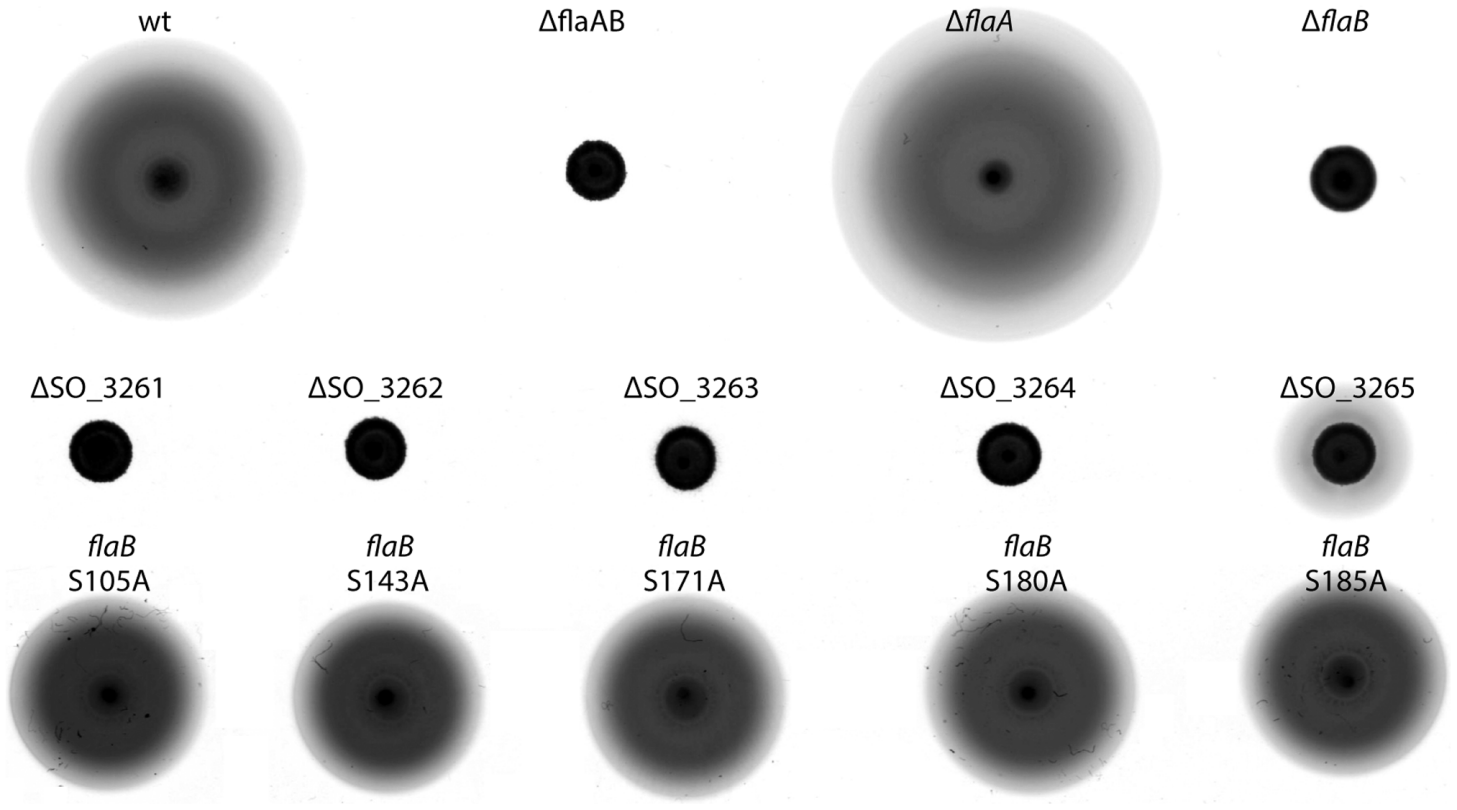
Phenotpyic analysis of *S. oneidensis* MR-1 flagellin mutants on soft agar. 3 µl of a corresponding strain culture was placed on LB soft agar (0.25%). The ability of cells to swim and navigate under those conditions is indicated by radial extension of the colonies due to flagella-mediated motility. Upper panel: Role of FlaA and FlaB in swimming. Middle panel: Analysis of deletion mutants in the flagellin modification gene cluster of *S. oneidensis* MR-1 (SO_3261– SO_3265 correspond to *sfmABCDE*). Lower panel: Phenotypic analysis of serine to alanine substitution mutations in the modified residues of FlaB.

To elucidate the role of the genes SO_3261-3265 in flagella-mediated motility, we introduced defined in-frame deletions into the corresponding genes. We then determined the swimming ability of the resulting mutants in soft agar plates and under the light microscope. Single deletions of each of the first four genes of the operon (SO_3261-3264) resulted in strains that were non-motile on soft agar plates (Fig. 2). Under the light microscope, only a few cells of ΔSO_3261, ΔSO_3262, and ΔSO_3264 were observed that were barely able to move. In contrast, a number of cells deleted in ΔSO_3263 were able to swim under those conditions. Accordingly, flagella staining revealed that almost all of the cells deleted in SO_3261, SO_3262 and SO_3264 lacked the flagellar filament (data not shown), while a visible flagellum was retained in the SO_3263 mutant. In contrast to the other four genes, a deletion of ΔSO_3265 did not lead to a loss of swimming motility in planktonic cultures and the cells still retained the flagellar filament. However, compared to the wild type, radial extension of ΔSO_3265 mutants on soft agar plates was significantly reduced. Reintroduction of the genes restored the wild-type phenotype, and none of the mutants was affected in growth (data not shown).

### SO_3261-SO_3265 are Involved in Flagellin Modification

**Figure 3:**
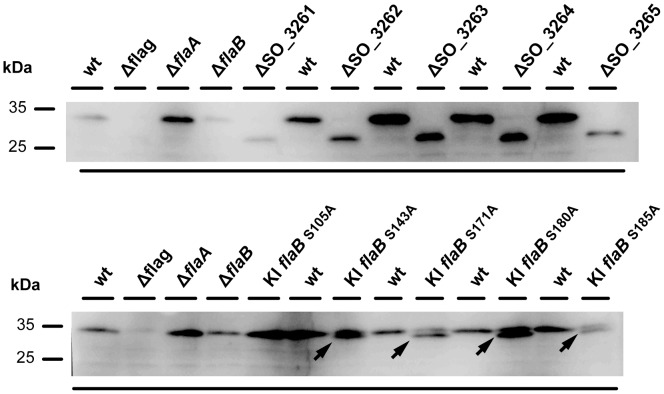
Analysis of the flagellin mass shift in response to mutations in FlaB or in genes of flagellin modification gene cluster. Displayed is a flagellin (FlaA and FlaB) immunostaining subsequent separation of a protein crude extract by SDS-PAGE (15% polyacrylamide) and Western transfer to a membrane. Upper panel: analysis of the migrating behavior of flagellin upon deletion of the genes SO_3261 to SO_3265 (*sfmABCDE*). Lower panel: migrating behavior of FlaB bearing serine to alanine substitutions in the indicated residues. The arrows point to bands exhibiting a significant mass shift. The upper band most likely corresponds to non-substituted FlaA.

The striking motility phenotype of the mutants, the inability of the mutants to assemble a fully functional flagellar filament, and the putative functions of the proteins encoded by some of the genes in the operon strongly suggested a role in modification of the flagellin. We therefore determined whether loss of the genes encoded in the operon results in a significant mass shift of the flagellins. To this end, whole protein extracts obtained from the wild type and corresponding mutants where separated by polyacrylamide gel electrophoresis (PAGE), and the flagellins were detected subsequent to Western transfer using antibodies raised against the flagellar filaments of *S. oneidensis* MR-1 (Fig. 3). Both FlaA and FlaB were readily detected at a position corresponding to a molecular mass of about 34 kDa as has been previously observed [33]. In contrast, the flagellins of the mutants lacking the genes SO_3261-3264 displayed a mass shift towards a position corresponding to a molecular mass of about 28 kDa, nicely matching the predicted mass of non-modified FlaA and FlaB. The mass shift of the flagellins obtained from the ΔSO_3265 mutant was also pronounced, but to a slightly lesser extent. Taken together, these findings strongly implicate a role for SO_3261-SO_3265 in modification of both FlaA and FlaB. Based on this, we will henceforth refer to this operon as *sfmABCDE* (for ***S*** *hewanella* ***f***lagellin ***m***odification).

### Characterizing the Chemical Nature of the Modification

So far, the studies on the flagellins of *S. oneidensis* MR-1 have provided evidence for a glycosylation of the flagellin protein, however, the corresponding sites as well as the nature of the modification remained unknown. To first analyze the chemical nature of the modification, we profiled the reduced, carboxyamidomethylated and trypsin-digested protein extract from *S. oneidensis* flagellin by various mass spectrometry techniques and analyzed the resulting data with *Proteome Discoverer* (Version 1.1, Thermo Scientific) using the *Sequest* algorism, searching the detected peptides against *S. oneidensis* flagellar sequences FlaA and FlaB. Both proteins share 89% identity and 93% similarity. In this way, we identified 67% of the peptides from FlaA and 82% of the peptides from FlaB. The results confirmed that both protein forms were present in the extract (Fig. S2). The signal intensities of peptides from FlaB were more abundant than those from Fla A. This is most likely because of the difference in protein concentration in the original flagellar extract. The FlaB is the major and FlaA is the minor subunit of the flagellar filament, as already predicted by phenotypical analysis.

**Figure 4:**
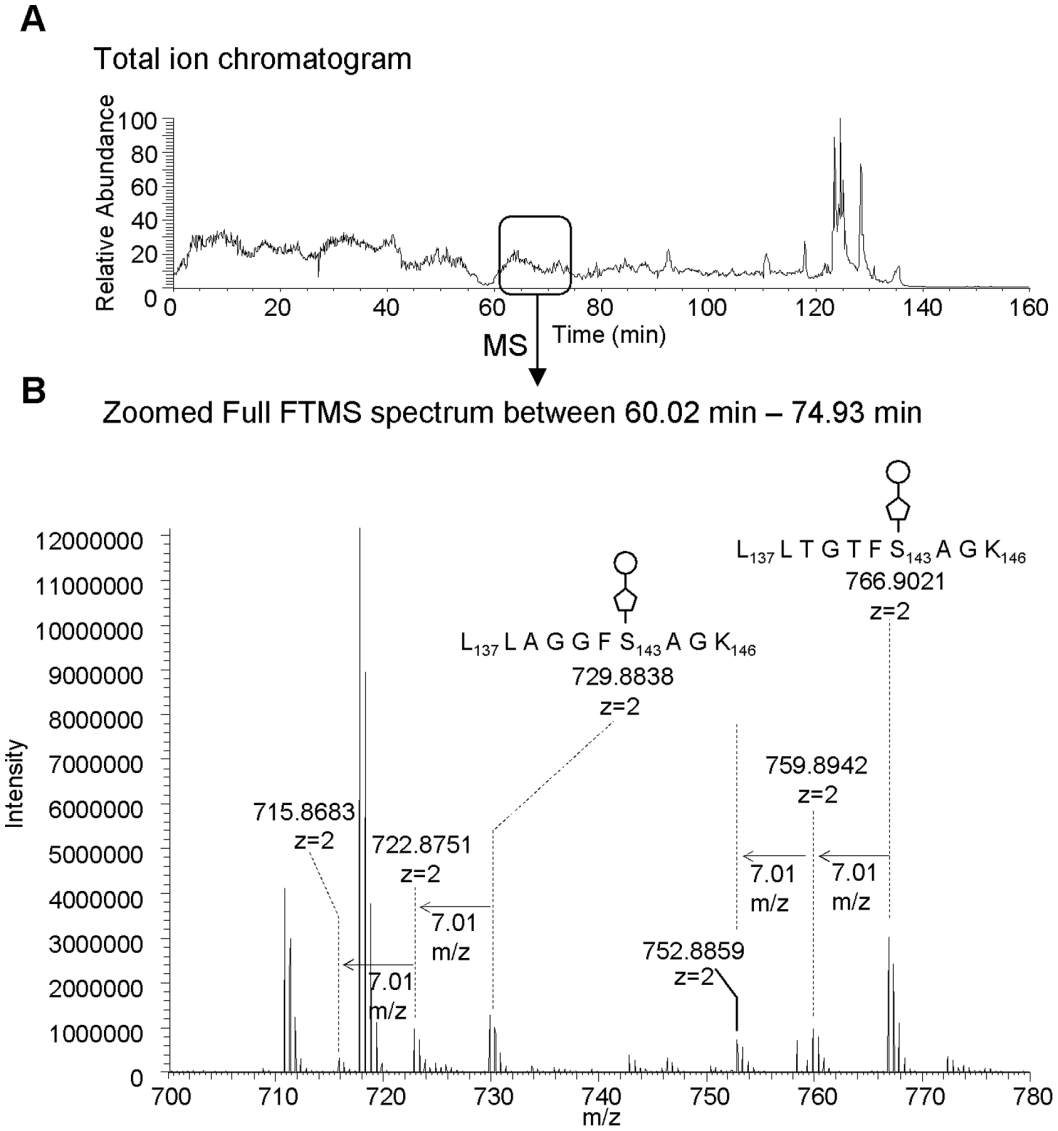
Analysis of tryptic digests of *S.oneidensis* flagellin preparations. A protein extract from flagella from *S.oneidensis* was reduced, carboxyamidomethylated and digested with trypsin then profiled by mass spectrometry. The chromatogram shown in **A** represents a total ion chromatogram of a LC-nano spray-MS/MS run. A series of modified peptides with peptide sequences L_137_LTGTFSAGK_146_ (from FlaA) or L_137_LAGGFSAGK_146_ (from FlaB) were eluted between 60 min to 75 min. A full FTMS spectrum between 60 min to 75 showed the accurate mass of the series of modified peptides in the flagella tryptic digest (A calibration of the instrument has been determined to be within 10 ppm accuracy).

**Figure 5:**
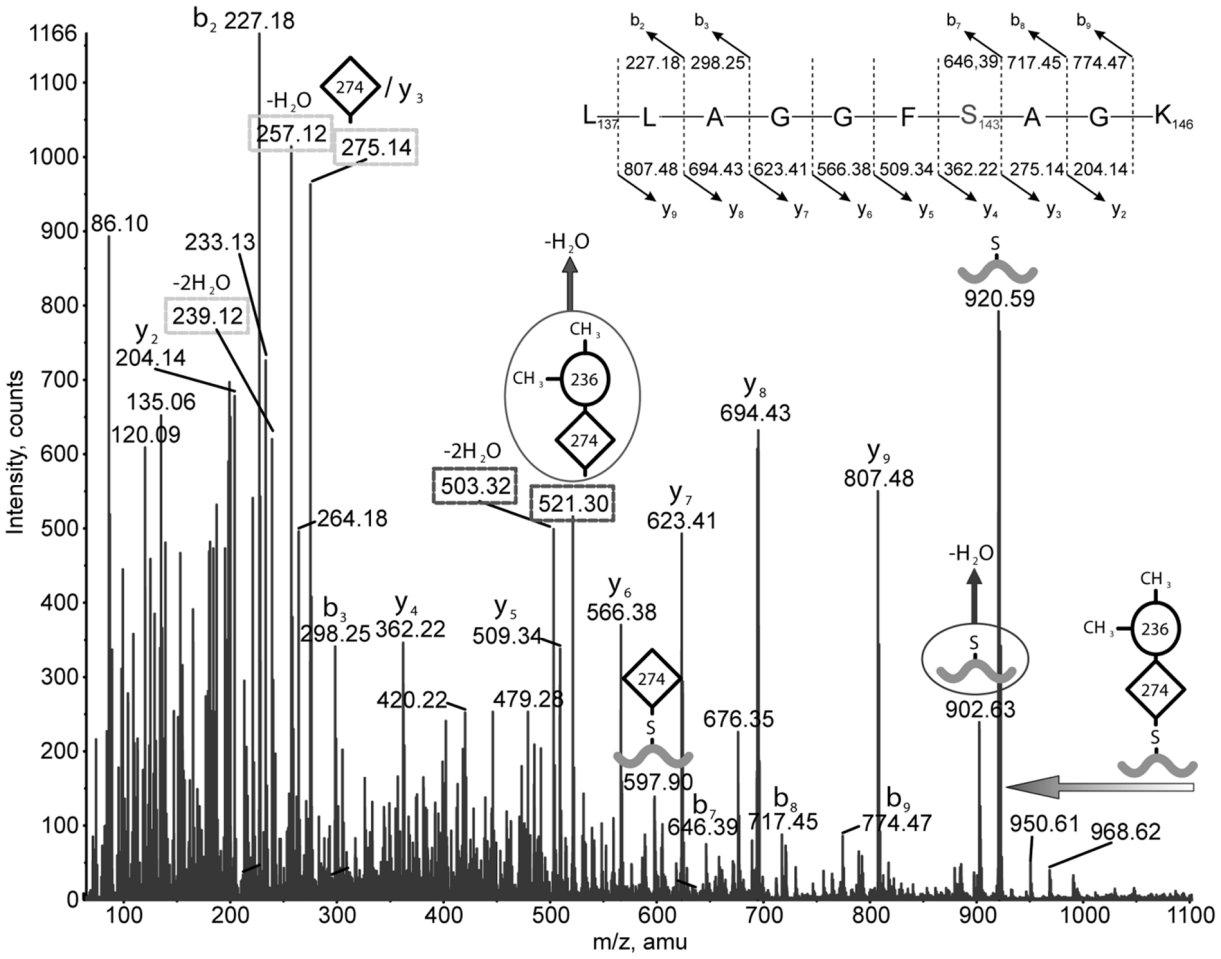
The MS/MS spectrum of 729.94^2+^ confirms the peptide sequence as LLAGGFSAGK. The sugar moiety has a mass of 538 Da. The predicted oxonium ion for this moiety (expected at m/z 539) is minor, but major signals are observed at m/z 521 and 503 for its mono and di-dehydrated forms, respectively. In contrast to the low abundance of m/z 539, a major signal is observed at m/z 275, corresponding to the oxonium ion of the m/z 274 moiety which is directly attached to the peptide. It is accompanied by a pair of intense signals at m/z 257 and 239 corresponding to its mono and di-dehydrated forms, respectively.

A manual search of the data indicated that most of the peptides not detected by the software were found to carry an unknown post translational modification (Fig. S2, Table 1). In the initial LC-MS/MS analysis of the tryptic digest we detected four modified tryptic peptides from each of the two flagellar proteins (Table 1). Figure 4 shows a portion of the full FTMS spectrum of two partially co-eluting modified peptides. The difference of the observed masses and the calculated peptide masses resulted in a mass of 538 Da for the main modification and 524 and 510 Da as minor components. Every mass spectrometric method we employed, including LC-QTOF-MS/MS (QStar), LC-Iontrap-MS/MS-CID, -ETD and -HCD (LTQ-orbitrap XL) and LC-IT-TOF (Shimadsu) detected these same components. Only LC-QTOF and IT-TOF detected additional mass differences of 274 Da and 520 Da. These ions were likely products of in-source fragmentation. The mass difference between 520 Da (in-source fragment) and 538 corresponds to loss of water, indicating that the 538 Da modification carries a functional group that can easily lose water, such as a hydroxyl or carboxyl group. The 274 Da mass difference suggested that the modifications have a 274 Da inner portion, from which the outer portion is easily cleaved in the source. This observation was confirmed by tandem mass spectrometry. Figure 5 shows the CID-MS/MS spectrum of the doubly charged ion at m/z 729.9, which corresponds to L_137_LAGGFSAGK_146_ with the 538-Da modification. The fragment ion at m/z 920 corresponds to the singly charged ion of the peptide backbone after loss of the post-translational modification. The ion at m/z 597 is the doubly charged ion of the peptide backbone with the 274-Da portion of the 538-Da modification. The ions at m/z 521 and m/z 503 result from loss of one or two moles of water from the oxonium ion of the 538-Da modification. Other observed peptides carrying the modification showed a similar fragmentation pattern. Overall, the LC-CID-MS/MS analysis suggested that the series of 510, 524 and 538 Da modifications are composed of two units, a 274 Da portion connected directly to the peptide, and a second portion of 236, 250, or 264 Da that is attached to the 274 Da moiety. The 14 mass differences observed between the 510, 524 and 538 Da modifications indicated different methylation stages, suggesting that the main modification of 538 Da carries at least two methyl groups on its 264 Da moiety. Also, the fact that the peptide backbone ion was singly charged, while the modified peptide ions, including the fragments that had lost their outer portions (e. g. m/z 597), were doubly charged suggested that the 274 portion carries a basic functional group which can be easily be protonated, such as an amino group. Neither the oxonium ion of the 264 Da moiety (expected at m/z 265), nor its de-methylated or dehydrated forms were detected in any glycopeptide carrying 510, 524 and 538 Da modifications, suggesting that the 264 Da moiety does not contain nitrogen. The MS/MS data from S_168_SLSVGSLGNTTSAAR_183_, A_164_SNKSSLSVGSLGNTTSAAR_183_, A_164_SNKTSLSVGALNNATSANR_183_ and T_168_SLSVGALNNATSANR_183_ peptides with excess masses above 538Da were very complex. This indicated that these tryptic peptides may contain multiple modification sites, which was later confirmed by LTQ-ETD-MS/MS analysis (described in the section below). Working with the assumption that there were two occupied sites, we were able to readily assign mass differences above 538 Da. For example, the 1048 Da mass excess was predicted as 510/538 Da combination and/or 524/524 Da combination, whilst two 538 Da increments gave 1076 Da, and these predictions were confirmed by LTQ-ETD-MS/MS analysis. For the four peptides, the only exception was the 1090 Da mass excess (Table 1). The modified peptides with 1090 Da excess mass yielded the same ions in the sugar fragment region as those observed for the 1076 Da modified peptide. The mass difference between 1090 Da and 1076 Da is 14 Da, which corresponds to a methyl group, suggesting that in addition to the two 538 Da modifications, the possibility of an additional methylation occurs in the peptide A_164_SNKTSLSVGALNNATSANR_183_ for FlaA, A_164_SNKSSLSVGSLGNTTSAAR_183_ for FlaB, respectively.

**Table 1:** Tryptic peptides with post translational modification(s) detected by nano-LC-QTOF-MS/MS.

Protein	Peptidesequence	Observedm/z	z	Mass excess(Da)
Fla A	D_94_LTIQSENGANSTADLVSIK_113_	*784.47*	*3*	*274* a
		1301.28	2	524
		1308.28	2	538
	T_168_SLSVGALNNATSANR_183_	1057.65	2	538
		*796,82*	*3*	*812*
		880.20	3	1062
		884,87	3	1076
	A_164_SNKTSLSVGALNNATSANR_183_	1022.61	3	1091
	L_137_LTGTFSAGK_146_	*634,91*	*2*	*274*
		*757,98*	*2*	*520*
		759,98	2	524
		766,97	2	538
Fla B	D_94_LTVQSENGANSSADLSALK_113_	*765.46*	*3*	*274*
		1266.25	2	510
		*847.53*	*3*	*520*
		1273.24	2	524
		1280.75	2	538
	S_168_SLSVGSLGNTTSAAR_183_	1023.62	2	538
		*769,47*	*3*	*798*
		*774,14*	*3*	*812*
		852,84	3	1048
		856,18	3	1058
		1285,77	2	1062
		1292,77	2	1076
	A_164_SNKSSLSVGSLGNTTSAAR_183_	1000.27	3	1091
	L_137_LAGGFSAGK_146_	*597,89*	*2*	*274*
		715,94	2	510
		*720,95*	*2*	*520*
		722.95	2	524
		729,94	2	538

a The ions shown italicized red fonts were observed from Time-of-flight (TOF) mass spectrometers, but not detected in the spectra generated from orbitrap and ion-trap mass spectrometers. It indicated that those are likely products of in-source fragmentation occurred within TOF mass spectrometers.

### Five Serines in FlaB and Four in FlaA are Modified

Furthermore, to determine the site of post-translational modification on flagella in *S. oneidensis*, we attempted the BEMAD procedure [36], but found the modification still attached by LC-MS/MS of the sequentially trypsin- and Glu-C-digested protein pellet, indicating an unusually base-stable bond between peptide and modification.

**Figure 6:**
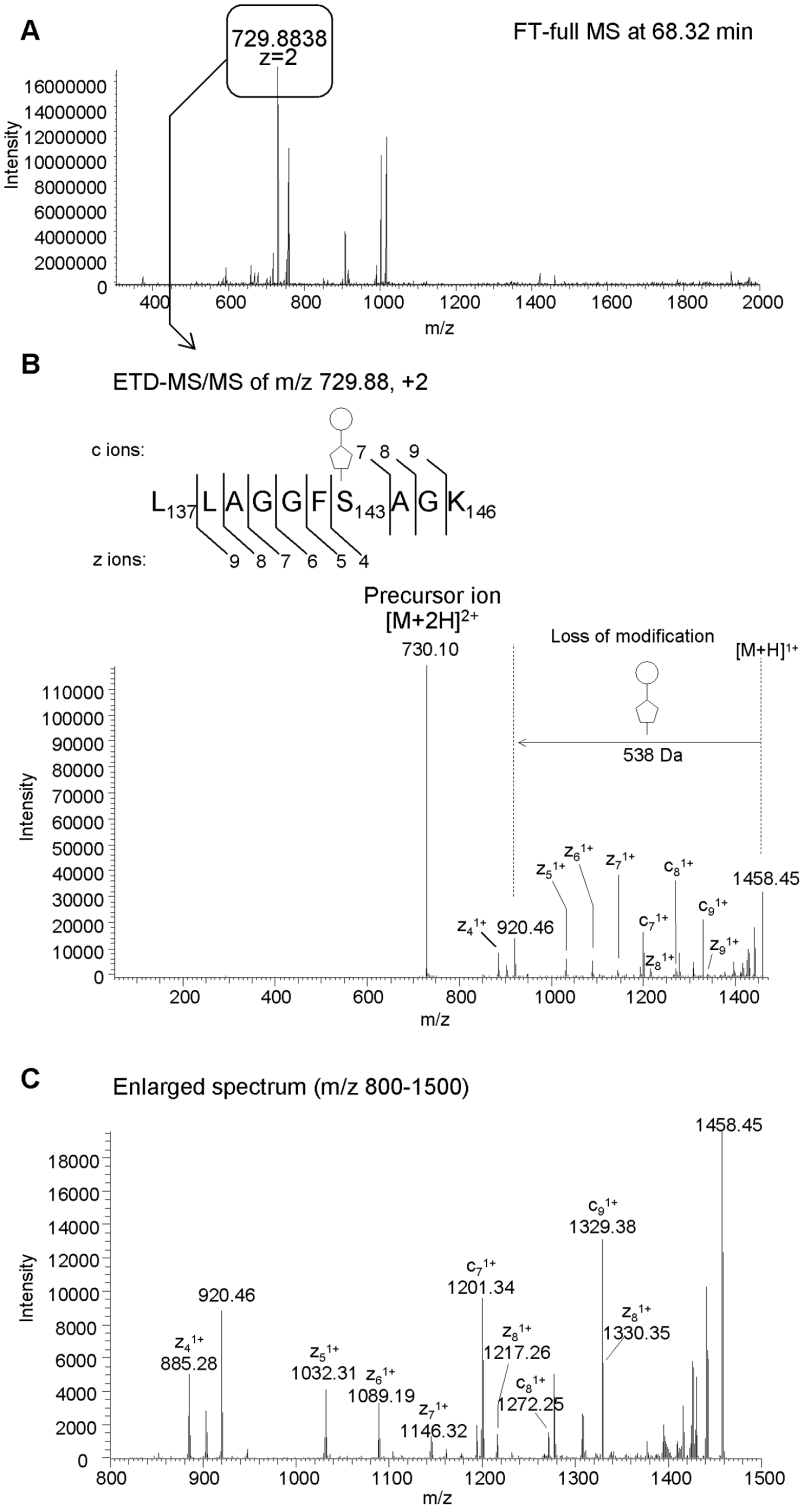
Mapping of the site of modification by LC-ETD-MS/MS. For the mapping of the site of modification, tryptic digest from *S.oneidensis* was profiled by LC-ETD-MS/MS. A modified peptide with a peptide sequence L_137_LAGGFSAGK_146_ plus a 538 Da modification was eluted at 68.32 min (A). An LC-ETD-MS/MS spectrum of peptide showed a series of c and z type fragments dues to peptide bond cleavages, indicated the site of modification at serine 143 (B). The spectrum (C) is an enlargement of spectrum B in the region m/z 800–1500. A cartoon structure of circle and pentagon in the spectrum represents 264 Da portion and 274 portion of the 538 Da modification.

We next turned to Electron Transfer Dissociation (ETD) for site mapping. ETD is a fragmentation method that effectively cleaves peptide bonds without breaking the bond between the modified amino acid and its modification, making this method ideally suited for site mapping experiments. We digested the flagellin protein with trypsin without removing the modifications and separated and analyzed the tryptic digest with an ETD-equipped LTQ-Orbitrap XL. ETD-MS/MS generated mainly c and z ions from peptide bond cleavages with minimum loss of the post-translational modifications, thus allowing us to determine the site of modification. Figure 6 represents the FT full MS and the ETD-MS/MS spectrum of a doubly charged modified L_137_LAGGFSAGK_146_ peptide ion at m/z 729.9. The ETD-MS/MS showed a series of c ions from c_7_ to c_9_, and of z ions from z_1_ to z_4_ of the glycopeptide, implicating Serine_143_ as the site of modification. The sites determined by LC-ETD-MS/MS, are summarized in Table 2. In FlaB, we found Ser_105_, Ser_143_, Ser_171_, Ser_180_ and Ser_185_ to be modified, and in FlaA, we detected at the same sites except Ser_105_. Since all the sites were serine residues, it is clear that the modification is *O*-linked and not *N-* or *C-*linked. Also, the data showed that the modification pattern is nearly identical in both protein forms, FlaA and FlaB. However, it cannot be completely ruled out that S_105_ on FlaA is also modified but escaped detection due to the lower abundance of FlaA in the mixture.

**Table 2:** Mapping of the site of unknown post translational modifications by LC-ETD-MS/MS.

Protein	Peptide sequence^a^	Observed m/z	z	Post translational modification^b^
Fla A	L_137_LTGTFS_143_@AGK_146_	766.9021	2	538
		759.8942	2	524
		752.8859	2	510
	T_168_SLS_171_@VGALNNATS_180_@ANR_183_	884.7679	3	538/538
	T_168_SLS_171_@VGALNNATS_180_@ANRAS_185_@SLAK_189_	937.7174	4	538/538/538
		1249.9576	3	
		934.2136	4	538/538/524
		1245.2833	3	
		930.7099	4	538/538/510538/524/524
		1240.6128	3	538/524/524
		927.2062	4	538/524/510524/524/524
		1235.9403	3	524/524/524
		1231.2672	3	524/524/510
		1226.5955	3	524/510/510
Fla B	D_94_LTVQSENGANS_105_@SADLSALKAEMDQLANEIDEIGK_128_	1054.5057	4	538
	L_137_LAGGFS_143_@AGK_146_	729.8838	2	538
		722.8751	2	524
		715.8683	2	510
	S_168_SLS_171_@VGSLGNTTS_180_@AAR_183_	862.0875	3	538/538
		857.4175	3	538/524
		852.7444	3	538/510524/524
		848.0726	3	524/510
		843.4006	3	510/510
	A_184_S_185_@SLK_188_	522.2722	2	538

**Mass spectral calibration was accurate within 10 ppm accuracy.**

a An @ indicates the site of post-translational modification determined by LC-ETD-MS/MS.

b The numbers listed in the column represents a modification or the combination of modifications attached on the site(s) on each modified peptide.

To further confirm the results obtained by ETD analysis we constructed mutants in FlaB, in which each of the identified serine residues was substituted with an alanine (S105A; S143A; S171A; S180A; S185A). The accordingly modified *flaB* genes were integrated into the chromosome to replace the native copy of *flaB*. On soft agar plates, all mutants bearing a single serine to alanine substitution were able to swim, albeit at a decreased level when compared to that of the wild type. To determine whether the serine substitutions also result in a mass shift of the flagellin, as would be expected by a loss of modified residues, we separated crude protein extracts obtained from the wild type and corresponding mutants by PAGE. The position of the flagellins was then detected after Western transfer by immunostaining. Two distinct bands were detected when FlaB carried substitutions in S_143_, S_171_, S_180_, and S_185_ (Fig. 3). The upper band most likely corresponds to non-mutated FlaA which is still fully modified, while the lower band indicates the mass shift of FlaB due to the substitution of the serine residues to alanine. Thus, taken together, the ETD and genetic analysis have identified at least four, more likely five, serine residues that are modified by O-linked glycosylation. The results demonstrate that none of the serine residues and their corresponding modification is absolutely required for assembly and function of the flagellar filament.

### Five Lysines on FlaB and One on FlaA are Methylated

**Figure 7:**
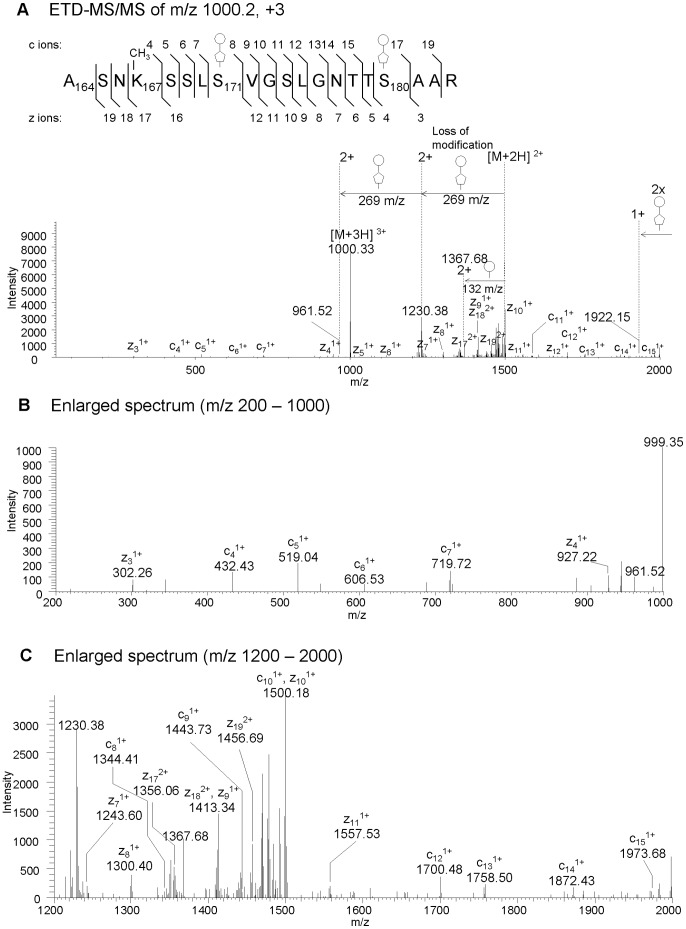
LC-ETD-MS/MS analysis of m/z 1000.2 A_164_SNK_167_@SSLS_171_@VGSLGNTTS_180_@AAR_183_ shows the sites of methylation and 538 Da modifications. LC-ETD-MS/MS data was analyzed for the site mapping of unknown modifications with 510-, 524- and 538 Da, as well as methylations on amino acids. The series of c and z type ions detected are assigned in the spectra A, B and C, as well as presented in a diagram located at the top of the figure. Spectra B and C are shown to present the enlarged spectrum of A in the regions m/z 200–1000 (B) and m/z 1200–2000 (C), respectively. The ions observed are the series of c and z ions, as well as those from loss of 538 Da modification(s). The series of c ions and z ions detected confirmed the peptide sequence, and indicated the site of methylation at Lysine 167 and the site of 538 Da modifications at Serine 171 and 180.

Moreover, we analyzed ETD-MS/MS data for the mapping of the site of possible methylations on amino acids, indicated by our primary LC-MS data analysis described above. An ETD MS/MS spectrum of m/z 1000.12 (3+), A164SNK167@SSLS171@VGSLGNTTS180@AAR, which includes the site of methylation at lysine 167, is shown in Figure 7 as an example. The ETD data generated was analyzed by *Proteome Discoverer 1.0* with the search criteria allowing 510-, 524- and 538 modifications and methylations. The data analysis by the software strongly indicated that the methylation occurs at Lysine 167, in addition to the 538 Da modifications on Serines 171 and 180. The modified peptides, including sites of methylation detected in this study are summarized in Table 3. The five lysines at positions 159, 163, 167, 188 and 189 on FlaB were methylated. On the other hand, only one methylated lysine, at position 159 of FlaA was found. We did not detect any other methylated lysines in FlaA, but this may have been because of the lower abundance of FlaA in the *S. oneidensis* flagellar extract.

**Table 3:** Mapping of the site of methylation by LC-ETD-MS/MS.

Protein	Peptide sequence^a^	Observed m/z	z	The site of modification(Post translational modification)^b^
Fla A	V_147_FQVGHQEGEDIK_159_@	500.58853	3	K_159_ (Methyl)
Fla B	N_147_FQVGAQDGEDIK_159_@	717.84760	2	K_159_ (Methyl)
	N_147_FQVGAQDGEDIK_159_@VTVK_163_@	621.32794	3	K_159_ (Methyl)
		625.99963	3	K_159_ (Methyl)
				K_163_ (Methyl)
	A_164_SNK_167_@SSLS_171_@VGSLGNTTS_180_@AAR	986.14813	3	K_167_ (Methyl)
				S_171_ (510 Da mod)
				S_180_ (524 Da mod)
		990.81970	3	K_167_ (Methyl)
				S_171_ (510 Da Mod)
				S_180_ (538 Da mod)
		995.49261	3	K_167_ (Methyl)
				S_171_ (524 Da mod)
				S_180_ (538 Da mod)
		1000.16376	3	K_167_ (Methyl)
				S_171_ (538 Da mod)
				S_180_ (538 da mod)
	A_184_S_185_@SLK_188_@K_189_@IDAAIK	595.00409	3	S_184_ (510 Da mod)
				K_188_ (Methyl)
				K_189_ (Methyl)
		599.67566	3	S_184_ (524 Da mod)
				K_188_ (Methyl)
				K_189_ (Methyl)
		604.34784	3	S_184_ (538 Da mod)
				K_188_ (Methyl)
				K_189_ (Methyl)
	K_189_@IDAAIK_195_	386.75037	2	K_189_ (Methyl)

**Mass spectral calibration was accurate within 10 ppm accuracy.**

a An @ indicates the site of post-translational modification determined by LC-ETD-MS/MS.

b The ( ) indicates that which modification attach on the corresponding site, which were determined by data analysis of the ETD data.

#### In-depth structural characterization of unknown-post translational modifications by high resolution MS analysis (LC-FT-MS/MS-CID and HCD)

We further analyzed tryptic peptides from *S. oneidensis* flagella by high resolution, high accuracy mass spectrometry to gain accurate precursor- and fragment mass of the unknown modification for a more in-depth structural characterization. We measured the precursor ions with the high accuracy LTQ Orbitrap-XL, featuring 60,000 resolution (FWHM at m/z 400) and detected the doubly charged ions of the modified peptides, L_137_LAGGFSAGK_146_ with a post translational modifications of 510, 524 or 538 Da, at m/z 715.8683, m/z 722.8751 and m/z 729.8838, respectively (Fig. 4B). The masses of singly charged ions are calculated as 1430.7293, 1444.7429 and 1458.7603 Da for the glycopeptides with 510, 524 or 538 Da modification, respectively. The theoretical mass of the singly charged peptide backbone, L_137_LAGGFSAGK_146_ is 920.5200 Da. The exact delta masses of the unknown modifications attached to the peptide are therefore 510.2093, 524.2299 and 538.2403 Da, respectively (with 10 ppm accuracy based on the mass accuracy of LTQ-Orbitrap XL).

**Figure 8:**
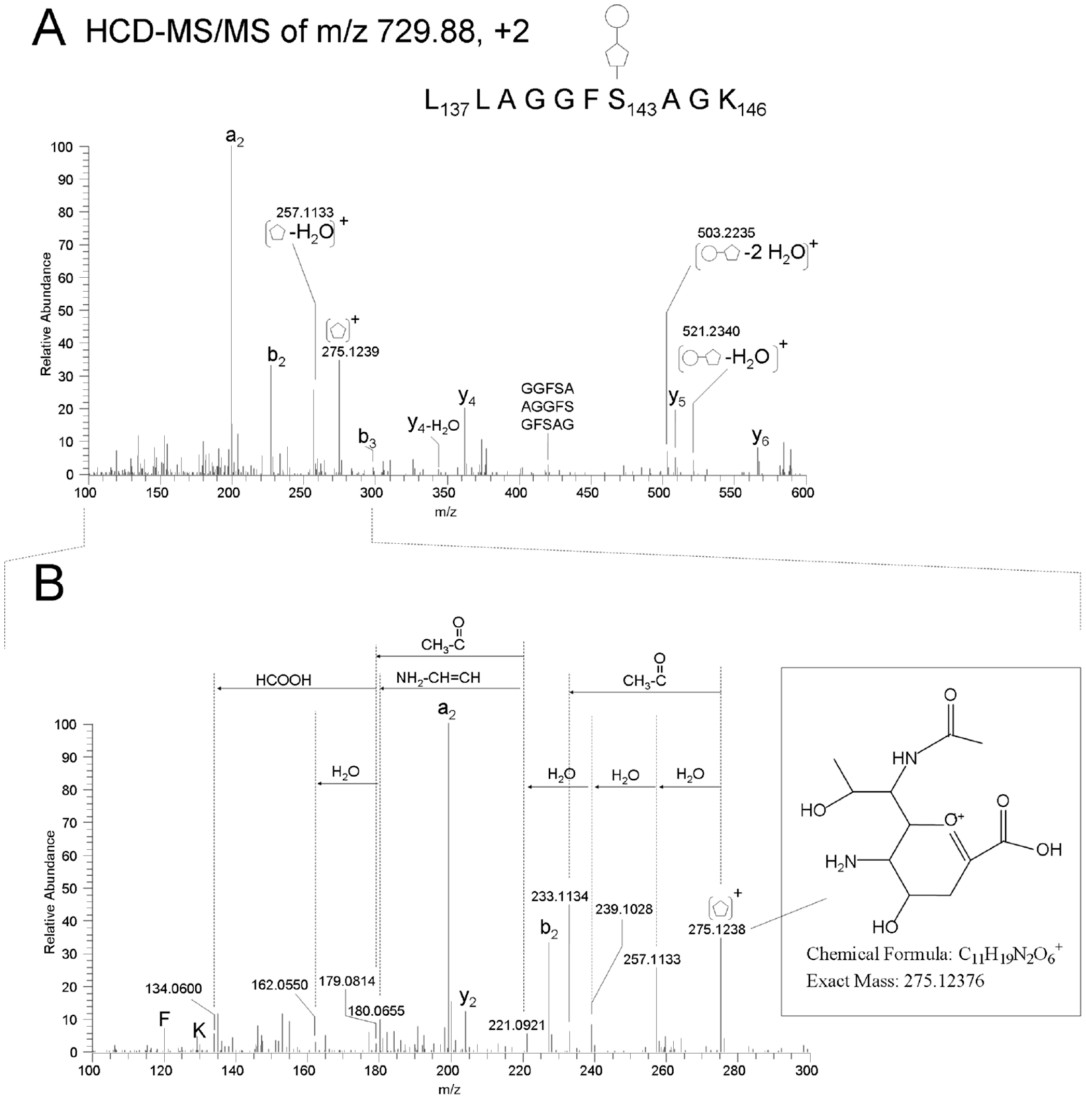
Analysis of the chemical nature of post-translational modification by LC-HCD-MS/MS. A HCD-MS/MS spectrum from L_137_LAGGFS_143_@AGK_146_ plus a 538 Da modification (@represents the site of modification), showed accurate mass of glycan-related ions correspond to dehydrated ions of the 538 Da modification, and oxonium ion from the 275 Da moiety of the 538 Da modification (A). The series of fragments from the 275 moiety were observed in the mass range between 100–300 m/z (B). An inset in the spectrum C shows a proposed structure of the 275 Da moiety. A pentagon and a circle cartoon in the figure shows 274 Da- and 250 Da moieties in the unknown modification, respectively.

We furthermore performed highly accurate HCD-MS/MS analysis on the tryptic peptides to determine the molecular formula of each portion of the unknown modifications. HCD fragmentation is available for the LTQ Orbitrap [37] where ions are fragmented in a collision cell rather than an ion trap and then transferred back through the C-trap for analysis at high resolution in the orbitrap. Compared with traditional ion trap-based collision-induced dissociation (CID), HCD fragmentation with orbitrap detection has no low-mass cutoff and a much higher resolution ion detection [38], thus allowing us to deduce a molecular composition from the observed mass of each fragment ion. Figure 8 shows an HCD-MS/MS spectrum from L_137_LAGGFSAGK_146_ with the 538 Da modification. We detected mostly y and b ions from the peptide backbone, which were all identified within 1 ppm mass accuracy, but also detected ions that did not match with any predicted peptide backbone fragments. These were most likely oxonium ions and other fragment ions of the modification. The 274 Da portion of the modification was observed as an oxonium ion at m/z 275.1238. The most likely formula for this oxonium ion is C_11_H_19_N_2_O_6_^+^ (theoretical m/z 275.12376). We also detected signals corresponding to one, two, and three losses of water from the 274-Da moiety with masses of 257.1133 Da (C_11_H_17_N_2_O_5_^+^, theoretical m/z 257.11320), 239.1028 Da (C_11_H_15_N_2_O_4_^+^, theoretical m/z 239.10263), and 221.0921 Da (C_11_H_13_N_2_O_3_^+^, theoretical m/z 221.09207). We also detected an ion resulting from the loss of an acetyl group from the 274-Da precursor at 233.1134 Da (C_9_H_17_N_2_O_5_^+^, theoretical m/z 233.11320) and three ions at 180.0654 Da (C_9_H_10_NO_3_^+^, theoretical m/z 180.06552), 162.0550 Da (C_9_H_8_NO_2_^+^, theoretical m/z 162.05495) and 134.0600 Da (C_8_H_8_NO^+^, theoretical m/z 134.06004). A modification with a similar fragmentation pattern has been described in *Campylobacter* flagella and was identified as 5,7-diacetamido-3,5,7,9-tetradeoxy-L-*glycero*-L-*manno*-nonulosonic acid (di-N-acetyl pseudaminic acid or Pse5Ac7Ac) [39]–[41]. Common pseudaminic acids found in campylobacter flagella, such as Pse5Ac7Ac, have two N-acetyl substitutions at the C-5 and C-7 positions. The theoretical mass of the oxonium ion from Pse5Ac7Ac is 317.1343 Da, which is 42 Da higher than that of the 275-Da moiety observed in *S. oneidensis*. The 42-Da difference corresponds to the lack of one N-acetyl group, and the theoretical mass of mono-N-acetyl pseudaminic acid agrees with our observed mass of m/z 275.1238 (Fig. 8; Table 4). The 274-Da portion of the 510- and 524-Da modifications showed a similar fragmentation pattern to that seen in the 538-Da modification (See Figures S3 and S4). The oxonium ions from the 510-, 524- and 538-Da modifications were themselves not detected in HCD-MS/MS in positive ion mode, most likely due to loss of the positive charge during the fragmentation. However, the oxonium ions that were observed in HCD analysis correspond to one and two losses of water from these ions. This allowed us to put forward a putative formula of the unknown modification (Table 4). Together with the delta mass calculated from the Orbitrap FT-MS data described above and the exact mass of the fragment ions observed in the HCD analysis, the 264 Da increment in the 538 Da modification was predicted to be C_11_H_20_O_7_ (as a neutral increment), whereas the 250 Da and the 236 Da moieties in the 524 Da and the 510 modifications were predicted to be C_10_H_18_O_7_ and C_9_H_16_O_7_, respectively (as neutral increments).

**Table 4:** Glycan related ions determined by high accuracy LC-HCD-MS/MS.

Observed m/z	Likely composition formula	Theoretical m/z	Note
275.1238^a^	C_11_H_19_N_2_O_6_^+^	275.12376	Detected as an oxonium ion of 274 Da moiety
521.2340^a^	C_22_H_37_N_2_O_12_^+^	521.23410	Detected as a dehydrated ion of 538 Da modification
507.2180^a^	C_21_H_35_N_2_O_12_^+^	507.21845	Detected as a dehydrated ion of 538 Da modification
493.2033^b^	C_20_H_33_N_2_O_12_^+^	493.20280	Detected as a dehydrated ion of 538 Da modification

**Mass spectral calibration was accurate within 1 ppm accuracy.**

a The mass was obtained from HCD-MS/MS spectrum of L_137_LAGGFS_143_@AGK_146_ (2+).

b The mass was obtained from HCD-MS/MS spectrum of L_137_LTGTFS_143_@AGK_146_ (2+).

#### IT-TOF and NMR

We prepared the tryptic digest of *S. oneidensis* flagellar protein in larger scale and purified tryptic peptides/glycopeptides from the sample by IT-TOF for further structural characterization of the modification by NMR. We reduced, carboxyamidometylated and digested with trypsin the resulting 8 mg of flagellar protein extract and separated the digest by LC-IT-TOF. We isolated the fraction containing mostly L_137_LAGGFSAGK_146_ with the 538-Da modification and analyzed it by NMR (the IT-TOF spectrum of the fraction is shown Fig. S5). The 2-D COSY NMR data (see Fig. S6) showed, besides the series of signals from amino acids, a spin system consistent with the H-3 (1.57 and 2.45 ppm) and H-4 (3.85 ppm) protons of β-pseudaminic acid. Unfortunately, the small coupling constants between H-4 and H-5 and between H-5 and H-6 [5], together with the spectral overlap of the amino acid signals prevented rigorous structure elucidation of the glycan. However, we did detect signals consistent with the presence of N-acetyl (∼2 ppm) and O-methyl (∼3.5 ppm) groups.

In summary, the 274 Da increment of the modification is most likely a nine carbon keto acid sugar (belonging to the nonulosonic acid family) containing two nitrogens and one N-acetyl group. This residue is located at the reducing end of the glycan and is attached to serine. By considering the previous findings from other groups regarding flagella modification, it is highly likely that this sugar is a member of the pseudaminic acid family. The chemical formulae of the 238-, 250- and 264-Da moieties were determined by mass spectrometry (Table 3). Oxonium ions related to post-translational modifications observed by an accurate HCD-MS/MS (Table 3) indicated the mass difference between the three components are 14.015 Da, corresponding to CH_2_ (theoretical m/z 14.01564). In ETD fragmentation analysis of modified peptides with 538 Da and 524 Da, we observed a neutral loss of 32 Da from the precursor glycopeptides, corresponding to loss of one methanol (CH_3_OH). This indicated that these modifications contain at least one *O-*methyl group, which was confirmed by NMR. These data support our prediction that the 14 mass differences observed between the 510, 524 and 538 Da modifications are due to different methylation stages. Since the 274-Da moiety does not carry any *O-*methyl group, we hypothesize the *O*-methylation(s) occur at the residue attached to the 274-Da moiety.

## Discussion

O-linked glycosylation has been demonstrated to be a common means of flagellin modification in many bacterial species. The number of glycosylated serine or threonine residues varies between different bacterial species and ranges from 19 residues in the heavily decorated FlaA protein of *C. jejuni* [41], [42] to two modified sites in *P. aeruginosa* FliC [43]. Here, we have demonstrated that the flagellin of *S. oneidensis* MR-1 is similarly modified at at least 4, more likely 5 serine residues in FlaB. The O-linkage occurs to a glycan of 274 Da that, according to our analysis, most likely is a pseudaminic acid with a single acetyl substitution. Such nonulosonic acid have previously been demonstrated to occur in flagellin modifications of, for example, *Campylobacter* sp. and *H. pylori* (reviewed in [17]). An additional moiety of 236 Da, 250 Da or 264 kDa is linked to the nonulosonic acid.

Generally, O-glycosylation of the flagellins is thought to occur before the flagellins are secreted through the flagellar type III export apparatus [16], [44]. In *Campylobacter* sp. and *H. pylori*, the corresponding glycosylation system consists of a set of enzymes that synthesize the appropriate sugar residues which are then linked to the flagellin peptide [21], [44]–[46]. In both *Campylobacter* as well as in *Helicobacter*, many of the proteins which are crucial for modification of the flagellin are encoded by the *pse* genes. Some of these proteins have been observed to localize to the flagellated cell pole of *C. jejuni*, implicating that the glycosylation machinery is directly linked to the flagellar basal body [44]. Recent work on the flagellins forming the filaments of the polar and lateral flagellar system in *Aeromonas hydrophila* identified homologs to *pseB* and *pseF* to be essential for glycosyslation [22]. Notably, a previous study identified a PseB homolog to be crucial for flagellin modification in *S. oneidensis* MR-1 [33], suggesting that a set of enzymes similar to Pse is required for that process in *Shewanella*. However, while homologs to PseB and PseC can be readily detected in *S. oneidensis* MR-1, clear orthologs to PseH, PseG, PseI, and PseF, which are also involved in synthesizing the glycan precursor in *Campylobacter*, are lacking in this species. The absence of these Pse enzymes implicates that formation of the carbohydrate requires additional proteins. In this work, we have identified a gene cluster, designated *sfmABCDE*, whose gene products are required for normal flagellin modification in *S. oneidensis* MR-1. In its N-terminal domain, SfmA exhibits homology to PseI (31% identity, 49% similarity). In addition, SfmC, which is predicted to function as a 3-oxoacyl-ACP-reductase, shares weak homologies to PtmA of *C. coli* (25%/43%), which has been implicated to play a role in flagellin modification in this species [47]. All other proteins encoded by the *sfm* operon differ significantly from factors that have been so far described to be involved in flagellin modification. SmfD is predicted to function as a methyl transferase, which is consistent with our observation of mass increments corresponding to one or two methyl groups being attached to the 236 Da moiety. Loss of the *smf* genes results in a significant mass shift of the FlaA and FlaB flagellins, strongly indicating that the corresponding gene products are crucial for glycan formation, their linkage to the serine residues in FlaC, or both. In *Campylobacter* sp., the transfer of the glycan to the flagellin is thought to be mediated by PseE and/or PseD, which have been demonstrated not to be required for the glycan formation cascade [48]. Notably, the central region of PseE shares 29% identity and 44% similarity to SO_3259, annotated as ‘motility accessory factor’ (MAF) and encoded by a gene which is located directly upstream of the *smf* operon, but likely not part of the operon. Similar homologies of SO_3259 occur to PseD (25%/40%). A second protein, SO_3273, also annotated as MAF, exhibits a similar degree of homology to PseE (22%/41%) and PseD (22%/35%). Thus, these two proteins might be another part of the *S. oneidensis* MR-1 flagellin glycosylation machinery and might function in glycan transfer to the flagellin. Notably, the mass shift of the flagellins in strains lacking SfmA-D appears to be similar, indicating that the modification needs to be fully assembled prior the transfer to the protein.

**Figure 9:**
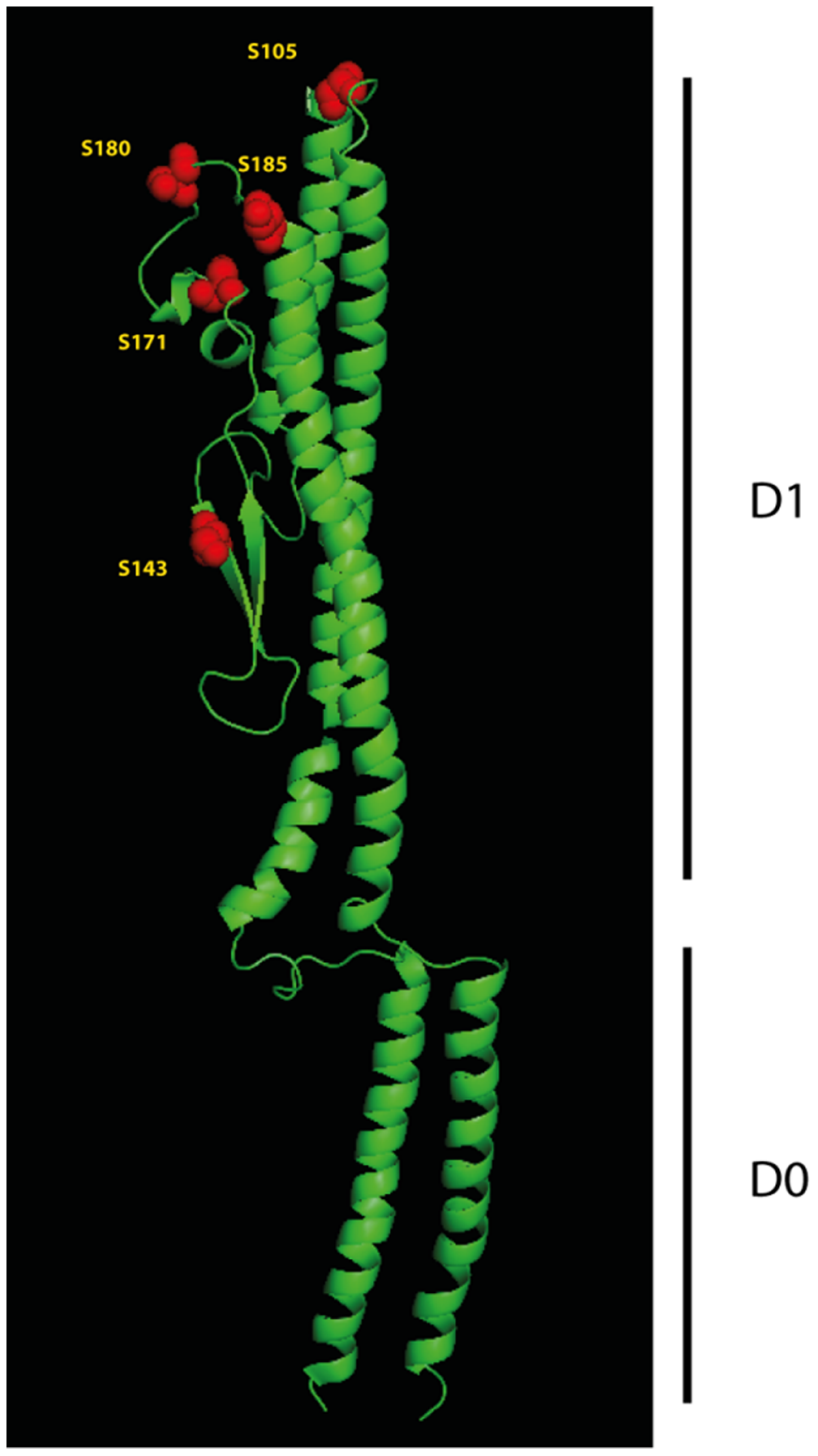
Predicted structure of *S. oneidensis* MR-1 FlaB. The structure of FlaB was modeled using the Phyre^2^ server. The position of predicted D0 and D1 domains required for assembly of the filament (in homology to *Salmonella* FlaC) is indicated to the right. The positions of the modified serine residues in FlaB are marked in red, the corresponding amino acid positions are indicated.

Commonly, the modified peptides reside in the central region of the flagellin, the part of the protein which is thought to be located at the surface of the flagellar filament [21], [22], [41]–[43]. Similarly, in *S. oneidensis* MR-1 FlaB the four serine residues are located between the highly conserved putative D0 and D1 regions, which are, based on the structure of *Salmonalla* FliC, predicted to be required for flagellin export and assembly [49]. Accordingly, structural modeling of *S. oneidensis* MR-1 FlaB using the Protein Homology/Analogy Recognition Enginge V 2.0 (Phyre^2^) [50] strongly suggested the presence of the D0 and D1 domains in a similar structure as those of *Salmonella* FliC, and that the serine residues of *S. oneidensis* FlaB, which were identified to be modified, are likely to be exposed to the surface of the filament (Fig. 9). One exception is residue S_105_ which is predicted to be located within the D1 region of FlaB. However, substitution of this residue to alanine did not result in a visible mass shift of the flagellin, suggesting that the identified modification might be different or might not occur in all flagellin molecules. Mutations that affect the ability to modify the residues lead to the loss of the flagellar filament, strongly indicating that the modification is crucial for its assembly or structural integrity. A comparison of the variable region of FlaA and FlaB proteins from a number of *Shewanella* species reveals a high degree of conservation even in this domain (Fig. S7). The serine residues that have been identified to be glycosylated in *S. oneidensis* MR-1 FlaB are conserved in a number of species, in particular those at the positions S_143_ and S_171_. Thus, it might be speculated that the flagellins of the corresponding *Shewanella* species are also modified. Notably, the *sfm* gene cluster has, so far, only been observed in a single additional species, *S. baltica* OS155. In contrast, orthologs to PseB/C and the MAF protein SO_3259 are readily identified in all *Shewanella* species with a flagellin homologous to *S. oneidensis* MR-1 FlaA and FlaB. Thus, it might be hypothesized that glycosylation of *Shewanella* FlaB (and likely FlaA) initiates with similar precursors such as pseudaminic (or a similar nonulosonic) acid, as described for other bacterial systems, while the final glycan modification occurs in a species-specific fashion. To further confirm the structure of the pseudaminic acid residue and to elucidate the structure of the 264-Da residue, we will remove the peptide by exhaustive protease digestion or β-elimination under harsher conditions. The purification of the glycan and its structural characterization are currently underway. Further analyses are also directed towards the question of whether the MAF proteins SO_3259 and SO_3273 are also part of the *S. oneidensis* glycosylation pathway. Our analysis also demonstrated that several lysine residues are methylated. So far, we have not determined whether or not function or if loss these methylations has any functional consequences. It is also unclear whether the *sfm* genes are involved in the observed lysine modifications. Thus, current studies address the question of the role of these additional modifications and whether the flagellins of other *Shewanella* species are similarly modified.

It should be noted that, while this manuscript was under review, another study on the posttranslational modification of the *S. oneidensis* flagellins was published [51]. Our present study nicely complements and expands these published findings due to the genetic analysis and a more detailed structural analysis.

## Materials and Methods

### Bacterial Strains, Growth Conditions, and Media

Bacterial strains and plasmids used in this study are summarized in Table 5 and Table 6, respectively. Cultivation of *E. coli* strains DH5α λpir and WM3064 were routinely carried out in LB medium at 37°C. Medium for the 2,6-diamino-pimelic acid (DAP)-auxotroph *E. coli* WM3064 was supplemented with DAP at a final concentration of 300 µM. *S. oneidensis* MR-1 was cultured in LB at 30°C. Solid media were prepared by adding 1.5% (w/v) agar. When appropriate, media were supplemented with 100 µg ml^−1^ ampicillin sodium salt, 50 µg ml^−1^ kanamycin and/or 10% (w/v) sucrose. For promoter induction of pGEX plasmids, IPTG was added to a final concentration of 1 mM in liquid medium.

**Table 5:** Strains used in this study.

strain	genotype	reference
***Escherichia coli***		
**DH5α λpir**	φ80d*lacZ*Δ*M15* Δ(*lacZYA-argF*)*U169 recA1 hsdR17 deoR thi-l supE44 gyrA96 relA1*/λpir	[64]
**WM3064**	*thrB1004 pro thi rpsL hsdS lacZ* ΔM15 RP4-1360 Δ(*araBAD*) 567Δ*dapA* 1341::[*erm pir*(wt)]	W. Metcalf, University of IIlinois,Urbana-Champaign
***Shewanella oneidensis***		
**S79**	MR-1 wild type	[65]
**S1059**	MR-1, Δ*sfmA* (SO_3261)	This study
**S843**	MR-1, Δ*sfmB* (SO_3262)	This study
**S1061**	MR-1, Δ*sfmC* (SO_3263)	This study
**S845**	MR-1, Δ*sfmD* (SO_3264)	This study
**S839**	MR-1, Δ*sfmE* (SO_3265)	This study
**S1021**	MR-1, Δ*flaA* (SO_3238)	This study
**S1020**	MR-1, Δ*flaB* (SO_3237)	This study
**S1334**	MR-1, Δ*pilMNOPQ* Δ*mshA* (SO_0281-5, SO_4105)	This study
**S2903**	MR-1, *flaB* S105A	This study
**S2904**	MR-1, *flaB* S143A	This study
**S2905**	MR-1, *flaB* S171A	This study
**S2906**	MR-1, *flaB* S180A	This study
**S2907**	MR-1, *flaB* S185A	This study

**Table 6:** Plasmids used in this study.

**pNPTS138-R6KT**	mobRP4^+^ ori-R6K sacB; beta-galctosidase fragment alpha;suicide plasmid for in-frame deletions or integrations; Km^r^	[55]
**pGP704Sac28Km**	mobRP4^+^ ori-R6K sacB; suicide plasmid for in-frame deletions; Km^r^	Chengyen Wu, unpublished
**pGEX-4T-1**	GST-fusion protein expression vector	GE Healthcare
**pGEX-4T1-SO3237-Nterm**	overexpression plasmid for purification of N-terminal domain of *flaB*	This study
**pGP704 Sac28 Km+KO-SO3261**	SO_3261 (*sfmA*) deletion fragment in pGP704Sac28Km	This study
**pGP704 Sac28 Km+KO-SO3262**	SO_3262 (*sfmB*) deletion fragment in pGP704Sac28Km	This study
**pGP704 Sac28 Km+KO-SO3263**	SO_3263 (*sfmC*) deletion fragment in pGP704Sac28Km	This study
**pGP704 Sac28 Km+KO-SO3264**	SO_3264 (*sfmD*) deletion fragment in pGP704Sac28Km	This study
**pGP704 Sac28 Km+KO-SO3265**	SO_3265 (*sfmE*) deletion fragment in pGP704Sac28Km	This study
**pNTPS R6KT+KO-SO3237**	SO_3237 (*flaB*) deletion fragment in pNPTS138-R6KT	This study
**pNTPS R6KT+KO-SO3238**	SO_3238 (*flaA*) deletion fragment in pNPTS138-R6KT	This study
**pNPTS138-R6KT-**Δ**pilM-Q**	SO_0281– SO_0285 (*pilMNOPQ*) deletion fragment in pNPTS138-R6KT	This study
**pNPTS138-R6KT-dmshA**	SO_4105 (*mshA*) deletion fragment in pNPTS138-R6KT	This study
**pNPTS138-R6KT-SOflaB-S105A**	*flaB*_S105A_ insertion fragment in pNPTS138-R6KT	This study
**pNPTS138-R6KT-SOflaB-S143A**	*flaB*_S143A_ insertion fragment in pNPTS138-R6KT	This study
**pNPTS138-R6KT-SOflaB-S171A**	*flaB*_S171A_ insertion fragment in pNPTS138-R6KT	This study
**pNPTS138-R6KT-SOflaB-S180A**	*flaB*_S180A_ insertion fragment in pNPTS138-R6KT	This study
**pNPTS138-R6KT-SOflaB-S185A**	*flaB*_S185A_ insertion fragment in pNPTS138-R6KT	This study

Km^r^, kanamycin resistance.

### Strains and Vector Constructions

DNA manipulations were performed using standard protocols or following the manufacturer’s instructions [52]. Genomic DNA was isolated from *S. oneidensis* MR-1 following essentially the protocol that was described earlier [53]. Kits for the isolation and purification of PCR products or plasmids were purchased from HISS Diagnostics GmbH (Freiburg, Germany). Enzymes were purchased from New England Biolabs (Frankfurt, Germany), Biozym Scientific GmbH (Hess. Oldendorf, Germany), and Fermentas (St Leon-Rot, Germany). Replicative plasmids were transferred into *E. coli* strains by transformation using chemically competent cells [54]. Generation of in-frame deletions or genomic introduction of *flaB* mutant versions into *S. oneidensis* MR-1 was performed using a kanamycin-resistance/sucrose-sensitivity counterselection procedure as described earlier [31], [55], [56]. The deletions were designed in a way that would only leave short gene segments of the 5′- and 3′-ends resulting in drastically truncated proteins (<30 amino acids). Successful deletions were verified by PCR. Substitution variants harboring point mutations in *flaB* (*flaB*-S105A, *flaB*-S143A, *flaB*-S171A, *flaB*-S180A or *flaB*-S185A) were constructed by overlap extension PCR using mismatched primer pairs. Markerless in-frame deletion mutants of *S. oneidensis* MR-1 leaving only short terminal sections of the target genes were constructed basically as reported earlier, using the suicide vectors pGP704Sac28KM (Chengyen Wu, unpublished) for construction of *sfmABCDE,* and pNPTS138-R6KT [55] for construction of *flaAB, pilMNOPQ* and *mshA* deletion plasmids. Primer sequences are available upon request. For an overexpression plasmid of the *flaB* N-terminal domain the corresponding fragment was amplified using appropriate primers prior to integration into pGEX-4T-1 (GE Healthcare). Overproduction of the protein and purification was carried out following the manufacturer’s instructions.

### Total RNA Extraction and Operon Mapping

For total RNA extraction of *S. oneidensis* MR-1 cells grown to the exponential phase under aerobic conditions in LB (OD_600_ of 0.5 to 0.7) were harvested by centrifugation at 4,600×*g* for 15 min at 4°C. Resulting cell pellets were immediately frozen in liquid nitrogen and stored at −80°C. Total RNA was extracted from S. oneidensis MR-1 cells by using the hot-phenol method [57]. Residual chromosomal DNA was removed by using Turbo DNA-Free (Applied Biosystems, Darmstadt, Germany) according to the manufacturer’s instructions. Extracted DNA-free total RNA was applied as the template for random-primed first-strand cDNA synthesis by using the RevertAid first-strand cDNA synthesis kit (Thermo Scientific/Fermentas, St. Leon-Rot, Germany) according to the manufacturer’s instructions. cDNA for Operon mapping was made using Superscript III Reverse Transcriptase (Invitrogen, Karlsruhe, Germany).

### Motility Assays

Motility assays were basically carried out on soft agar plates as described before [58], [59]. 3 µl of a liquid culture of the corresponding strain were spotted on plates containing LB medium solidified with an agar concentration of 0.2% (w/v). For direct comparison of strains, the aliquots were always spotted onto the same plate. The plates were incubated at 30°C for an appropriate amount of time, subsequently scanned, and the radial extension of the cells from the center of the colony was determined.

### Flagellar Filament Preparation

For preparation of native flagellar filaments and subsequent mass spectroscopic analyses a protocol was used similar to what was described before [60]. Cells of a strain lacking cell appendages other then flagellar filaments (Δ*pilM-Q* Δ*mshA*) were incubated to the exponential phase and harvested at 13.000×g prior to resuspenision in TBS (0.02 M Tris-HCl, 0.15 M NaCl, pH 7.5). Flagellar filaments were sheared off the cell bodies by harsh vortexing at 4°C for 1 hour. The filaments were subsequently separated from the cell bodies through slow centrifugation at 6000×g at 4°C, giving rise to a supernatant containing the soluble flagellar filaments. Ultra-centrifugation at 120,000×g was used to harvest and concentrate the flagellar filaments for subsequent procedures. Flagellar pellets were resuspended in TBS and frozen at −20°C for long-term storage.

### Sample Preparation for the Site and the Chemical Nature of Post Translational Modification (PTM) Analysis by Mass Spectrometry

The sample was reduced with 25 mM dithiothreitol (DTT) for 1 hr at 55°C and carboxyamidomethylated with 90 mM iodoacetamide in the dark for 45 min. The sample was dialyzed with a 4 kDa cut-off membrane (Millipore) against nanopure water at 4°C overnight to remove salts and then dried in a Speed Vac. For the sample analysis/purification by LC-ETD/HCD-MS/MS and IT-TOF the sample was digested with Trypsin (Promega, sequence grade for ETD/HCD analysis; Sigma (T8003) for IT-TOF). For O-glycosylation site mapping by BEMAD [36], the sample was suspended in 1% triethlamine, 0.1% NaOH and 0–20% of ethanol (peptides 20%, proteins 0%) and 10% DTT. The reaction was incubated at 45°C for 4 hours. The sample was then desalted, resuspended in 50 mM NH_4_HCO_3_ and digested with Trypsin (Promega), at 37°C overnight. A small aliquot of the tryptic digest was profiled by LC-MS/MS and the rest of the digest was further digested with Glu-C (Roche) at 25°C overnight and dried by either a speed vacuum concentrator or lyophilization. The dried sample was resuspended with solvent A (0.1% formic acid in water) and passed through a 0.2 *µ*m filter (Nanosep, PALL) prior to mass spec analysis.

### Analysis of Tryptic Digest from *S.oneidensis* Flagella by nanoLC-ESI-QTOF-MS/MS

Tryptic digests were analyzed by nano-LC-ESI-QTOF-MS/MS using a reverse-phase nano-HPLC system (Dionex, Sunnyvale, CA) connected to a quadrupole TOF mass spectrometer (Q-STAR Pulsar I, MDS Sciex). The digests were separated by a binary nano-HPLC gradient generated by an Ultimate pump fitted with a Famos autosampler and a Switchos microcolumn switching module (LC Packings, Amsterdam, The Netherlands). An analytical C18 nanocapillary (75 m inside diameter × 15 cm, PepMap) and a micro precolumn C18 cartridge were employed for on-line peptide separation. The digest was first loaded onto the precolumn and eluted with 0.1% formic acid (Sigma) in water (HPLC grade, Purite) for 4 min. The eluant was then transferred onto an analytical C18 nanocapillary HPLC column and eluted at a flow rate of 150 nL/min using the following gradient of solvent A [0.05% (v/v) formic acid in a 95∶5 (v/v) water/acetonitrile mixture] and solvent B [0.04% formic acid in a 95∶5 (v/v) acetonitrile/water mixture]: 99% A from 0 to 5 min, 99 to 90% A from 5 to 10 min, 90 to 60% A from 10 to 70 min, 60 to 50% A from 70 to 71 min, 50 to 5% A from 71 to 75 min, 5% A from 75 to 85 min, 5 to 95% A from 85 to 86 min, and 95% A from 86 to 90 min. Data acquisition was performed using Analyst QS software with an automatic information-dependent-acquisition (IDA) function.

### LC- CID-MS/MS for the Site of O-glycosylation Mapping by BEMAD

For the site of O-glycosylation mapping by BEMAD, LC-MS/MS analysis was performed on an LTQ Orbitrap XL mass spectrometer (ThermoFisher) equipped with a nanospray ion source. The filtered sample was loaded onto a nanospray tapered capillary column/emitter (360×75×15 µm, PicoFrit, New Objective, Woburn, MA) self-packed with C18 reverse-phase resin (10.5 cm, MICHROM Bioresources, Inc.) in a nitrogen pressure bomb at 1,000 psi and then separated via a 160 min gradient of increasing solvent B (80% acetonitrile, and 0.1% formic acid in water) at a flow rate of ∼500 nL/min directly into the mass spectrometer.

The LTQ was run in automatic mode collecting an MS scan (Full FTMS at 300–2000 m/z) followed by data dependent MS/MS scans of either the six most intense ions from a calculated parent mass list or the six most abundant precursor ions if listed masses were not found. The parent masses were calculated from the tryptic, or trypsin/Glu-C peptides with potential O-glycosylation sites allowed for mass increase of 136.0017 for the DTT-modification. The resulting data were analyzed manually as well as by software (Proteome Discoverer 1.1, Thermo Scientific). For the data analysis by the software, the data was searched against the *S. oneidensis* MR-1 protein sequences SO_3237 and SO_3238, using the TurboSequest algorithm. The SEQUEST parameters were set to allow 50.0 ppm of precursor ion mass tolerance and 0.8 Da of fragment ion tolerance with monoisotopic mass. Digested peptides were allowed with up to three missed internal cleavage sites, and the differential modifications of 57.0215 Da, 15.9949 Da and 136.0017 Da were allowed for alkylated cysteine, oxidation of methionines and DTT-modified serine and threonine, respectively. The peptides identified by the software were further filtered based on XCorr value [61], 2.0 for the singly charged ions, 2.5 for the doubly charged ions and 2.75 for the triply charged ions.

### Analysis of Post Translational Modifications by LC-ETD- and HCD-MS/MS

In order to map and analyze post translational modifications, an LC-MS/MS analysis was performed on a ThermoFisher LTQ-Orbitrap XL equipped with a nanospray source and ETD and HCD fragmentation capabilities. The sample was loaded onto a C18 self-packed capillary column and separated by the same solvent and the same gradient program that was used for the BEMAD-derivative described above. An instrument method was used to collect a full mass spectrum, followed by fragmentation from a parent mass list or, if absent, the most abundant ions present in the spectra, via HCD and ETD. Dynamic exclusion was set at 1 for 30 seconds. Fourier Transform mode was used to fragment peptides utilizing 38% Higher Energy collision Dissociation (HCD) with a 2.5-m/z isolation width and a resolution of 15,000 (at 400 m/z). Ion Trap mode was used to fragment peptides utilizing Electron-Transfer Dissociation (ETD) for 100 ms with supplemental activation energy and a 3.0-m/z isolation width. The resulting data was analyzed manually as well as by the software (Proteome Discoverer 1.1, Thermo Scientific) using the TurboSequest algorithm. For the analysis by the software, a precursor ion mass tolerance was allowed up to 10.0 ppm and fragment ion tolerance was set up to 0.8 Da with monoisotopic mass. Digested peptides were allowed with up to three missed internal cleavage sites, and the differential modifications of 57.0215 Da, 15.9949 Da and 14.0156 Da for alkylated cysteine, oxidation of methionines, and mwthylation respectively. Furthermore, the accurate delta mass of post translational modifications were calculated from the full mass data obtained in Fourier transform mode and used for making the parent mass list for selected MS/MS analysis and data analysis by the software.

### Purification of Tryptic Peptides with Post Translational Modification by IT-TOF

Purification of tryptic modified peptides was performed on an IT-TOF (Shimadzu). The tryptic digest was filtered, and 100 µL was injected via autosampler. Separation was accomplished by a C18 reversed-phase HPLC Column (Vydac 218TP54; 5 µm, 4.6 mm i.d. ×250 mm), and the sample was eluted by a gradient elution with solvent A (0.1% formic acid in water) and B (80% acetonitrile, and 0.1% formic acid in water) using a 70 min gradient of increasing solvent B at a flow rate of 0.7 mL/min. The eluent was split at a 1∶300 ratio. The smaller portion was measured by full mass analysis in positive ion mode, followed by MS/MS analysis of the six most abundant ions. The rest was monitored by PDA with the range of 190 nm–600 nm and then collected in a fraction collector. The fraction collector was set to collect 1 mL/fraction. The fractions containing modified peptide were dried in a speed vacuum concentrator and analyzed by NMR.

### NMR

The isolated modified peptides were deuterium-exchanged by lyophilization from D_2_O (99.9%D, Aldrich), dissolved in 270 µL D_2_O (99.96%D, Cambridge Isotope), and placed into a 5-mm NMR tube with D_2_O-matched magnetic susceptibility plugs (Shigemi). One-dimensional proton and 2-D gradient-enhanced COSY NMR spectra were acquired at 25°C on a Varian Inova 800 spectrometer, equipped with a cryogenic triple-resonance probe. Spectral width was 7184 Hz, and 256 increments with 8 scans each were collected. Acquisition time was 150 ms, resulting in 1078 datapoints per FID, which were zero-filled to 2048 final points using the Mestre-C software. The data was processed with a sinebell function in both dimensions and linear prediction (119 basis points, 8 coefficients) in F1. Chemical shifts were referenced to internal acetone (δ = 2.218 ppm).

### Flagellar Staining

Staining of flagellar filaments was carried out as described earlier [62]. 5 µl of a culture of the corresponding strain at an appropriate growth phase in LB medium was used for visualization. Microscopy and image acquisition was carried out on an Axio Imager.M1 microscope (Zeiss, Wetzlar, Germany) with a Zeiss Plan Apochromate 100x/1.4 differential interference contrast or phase contrast objective. The MetaMorph® Microscopy Automation & Image Analysis Software was used for image analysis and processing.

### Immunoblot Analysis

To determine the molecular mass of flagellin protein, lysates from exponentially growing LB cultures were obtained for Western blot analyses. Appropriate cells were harvested by centrifugation and resuspended in sample buffer [63] according to an OD_600_ of 10. Subsequently, samples were heated at 99°C for 5 min, and stored at −20°C. 10 µl of sample were resolved by SDS-PAGE using 15% polyacrylamide gels. Proteins were transferred to nitrocellulose Immobilon-P membrane (Millipore, Schwalbach) by semidry transfer. For detection of flagellin FlaA and FlaB, polyclonal antibodies were used that were raised against the N-terminal conserved region of FlaB (Eurogentec Deutschland GmbH, Köln, Germany) in the dilution of 1∶600. Secondary anti-rabbit immunoglobulin G-horseradish peroxidase antibody was used at a dilution of 1∶20 000 for detection of N-terminus FlaB antibodies. Signals were detected using the SuperSignal® West Pico Chemiluminescent Substrate (Thermo Scientific, Schwerte, Germany) and documented using the CCD System LAS_4000 (Fujifilm, Düsseldorf, Germany).

## Supporting Information

Figure S1 **RT PCR analysis of the putative** ***sfm*** **operon start.** The upper panel displays the genetic organization of *sfmABC* and the upstream genes SO_3260 and SO_3259. The positions of the expected PCR products obtained from cDNA are indicated above. Lower panel: RT PCR analysis. Lane I: control with chromosomal DNA as template; lane II, cDNA template; lane III, control assay without Reverse Transcriptase; lane IV, no template.
(PDF)

Figure S2 **Protein identification in** ***S. oneidensis*** **flagellar extract by mass spectrometry.** The flagella extract of S. oneidensis was reduced, carboxyamidomethylated and digested with trypsin, and profiled by various mass spectrometry techniques. The figure represents the data from LTQ-orbitrap-XL (LC-NSI-CID-MS/MS). The resulting data was analyzed by Proteome Discoverer (Version 1.1, Thermo Scientific) using the Sequest algorism, and the detected peptides were searched against S. oneidensis flagella sequences SO_3237 (FlaB) and SO_3238 (FlaA). The peptides identified by the software are highlighted in green. 82% of the peptides from SO_3237 (FlaB) and 67% of the peptides from SO_3238 (FlaA) were identified from the protein extract.
(PDF)

Figure S3 **HCD-MS/MS spectrum from L_137_LAGGFS@AGK_146_ plus a 522 Da modification.** (@represents the site of modification). The HCD-MS/MS spectrum showed accurate mass of glycan-related ions correspond to dehydrated ions of the 522 Da modification, and oxonium ion from the 274 Da moiety of the 522 Da modification (A). The series of fragments from the 274 moiety were observed in the mass range between 100-300 m/z (B). An inset in the spectrum C shows a proposed structure of the 274 Da moiety (observed m/z of 274 Da moiety as an oxonium ion is m/z 275). Pentagon cartoon and grayed circle cartoons in the figure represents 274 Da- and 250 modifications, respectively.
(PDF)

Figure S4 **HCD-MS/MS spectrum from L_137_LTGTFS@AGK_146_ plus a 510 Da modification** (@represents the site of modification). The HCD-MS/MS spectrum showed accurate mass of glycan-related ions correspond to dehydrated ions of the 510 Da modification, and oxonium ion from the 274 Da moiety of the 510 Da modification (A). Figure 3 B represents the zoomed HCD spectrum between m/z 100 to 300. It should be noted that the signals observed from the peptide with 510 Da were very low compared to those with 524 Da or 538 Da modifications, dues to low abundance of the 510-Da modification occurs in the flagella. However, we detected main fragment ions from 274-Da modification. An inset in the spectrum C shows a proposed structure of the 274-Da moiety (observed m/z of 274 Da moiety as an oxonium ion is m/z 275). Pentagon cartoon and black-colored circle cartoons in the figure represent 274 Da- and 236 modifications, respectively.
(PDF)

Figure S5 **Glycopeptide purification by IT-TOF.** The tryptic digest of *S. oneidensis* flagellar protein was prepared in larger scale and tryptic peptides/glycopeptides were purified from the sample by IT-TOF for further structural characterization of the modification by NMR. The sample elution was monitored on-line by IT-TOF mass spectrometry. The figure represents the spectra of the fraction 15, containing mostly L_137_LAGGFSAGK_146_ with the 538-Da modification, which was analyzed by NMR.
(PDF)

Figure S6 **2D-NMR analysis of a glycopeptide fraction purified from** ***S.oneidensis***. Two-Dimensional gradient-enhanced COSY NMR spectrum of Fraction 15, containing mostly L137LAGGFSAGK146 with the 538-Da modification, run at 800 MHz and 25 °C. The dotted lines connect the two H-3 protons and the H-4 proton of the nonulosonic acid detected in MS.
(PDF)

Figure S7 **Alignment of the variable regions of** ***Shewanella*** **flagellins.** Variable regions of *S. oneidensis* MR-1 FlaA and FlaB (aa 101-189) were aligned to corresponding flagellin sequences of *Shewanella* spp. harbouring flagellins with a similar domain structure (comprising between 260 and 280 aa). Modified residues in FlaB are marked with arrows and in red. At some positions, several flagellins have a threonine instead of a serine residue (marked in green) that might be similarly modified. Invariable residues are highlighted in yellow, and positions with a single amino acid variation are marked in grey. Locus tag coding: Swoo, *S. woodyi*; Ssed, *S. sediminis*; Shew, *S. loihica*; Shal, *S. halifaxensis*; Sden, *S. denitrificans*; Sputw3181, *S.* sp. W3-18-1; Shewana3, *S.* sp. ANA-3; Sbal183, *S. baltica* OS183; Shew185, *S. baltica* OS185; Sput200, *S. putrefaciens* 200.
(PDF)
