# Supplementary material for: Analyzing the Modification of the Shewanella oneidensis MR-1 Flagellar Filament
Source: PLoS One. 2013 Sep 6;8(9):e73444. doi: 10.1371/journal.pone.0073444 (PMC3765264; doi:10.1371/journal.pone.0073444)
Supplement: Figure S1 — RT PCR analysis of the putative sfm operon start. The upper panel displays the genetic organization of sfmABC and the upstream genes SO_3260 and SO_3259. The positions of the expected PCR products obtained from cDNA are indicated above. Lower panel: RT PCR analysis. Lane I: control with chromosomal DNA as template; lane II, cDNA template; lane III, control assay without Reverse Transcriptase; lane IV, no template. (PDF) [file pone.0073444.s001.pdf]

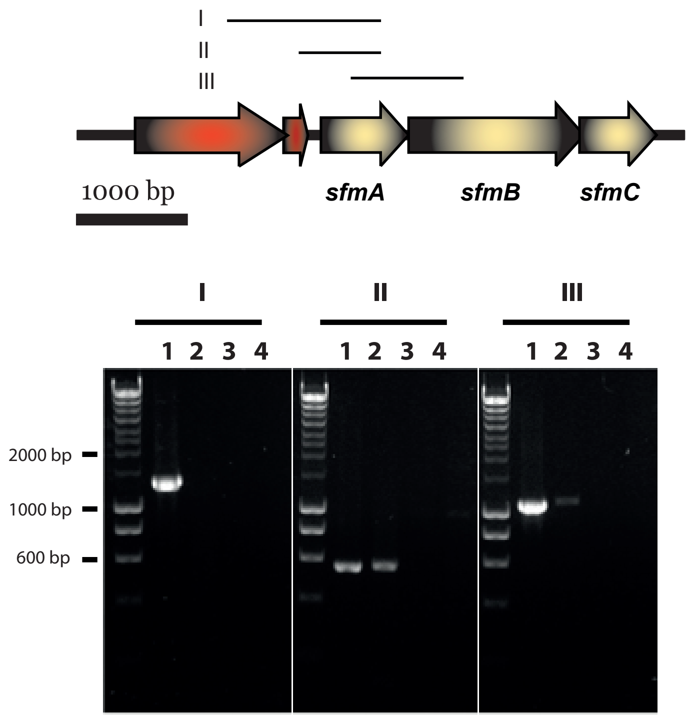

Supplemental Figure 1: RT PCR analysis of the putative *sfm* operon start. The upper panel displays the genetic organization of *sfmABC* and the upstream genes SO_3260 and SO_3259. The positions of the expected PCR products obtained from cDNA are indicated above. Lower panel: RT PCR analysis. Lane I: control with chromosomal DNA as template; lane II, cDNA template; lane III, control assay without Reverse Transcriptase; lane IV, no template.
