# Supplementary material for: Analyzing the Modification of the Shewanella oneidensis MR-1 Flagellar Filament
Source: PLoS One. 2013 Sep 6;8(9):e73444. doi: 10.1371/journal.pone.0073444 (PMC3765264; doi:10.1371/journal.pone.0073444)
Supplement: Figure S2 — Protein identification in S. oneidensis flagellar extract by mass spectrometry. The flagella extract of S. oneidensis was reduced, carboxyamidomethylated and digested with trypsin, and profiled by various mass spectrometry techniques. The figure represents the data from LTQ-orbitrap-XL (LC-NSI-CID-MS/MS). The resulting data was analyzed by Proteome Discoverer (Version 1.1, Thermo Scientific) using the Sequest algorism, and the detected peptides were searched against S. oneidensis flagella sequences SO_3237 (FlaB) and SO_3238 (FlaA). The peptides identified by the software are highlighted in green. 82% of the peptides from SO_3237 (FlaB) and 67% of the peptides from SO_3238 (FlaA) were identified from the protein extract. (PDF) [file pone.0073444.s002.pdf]

FlaA

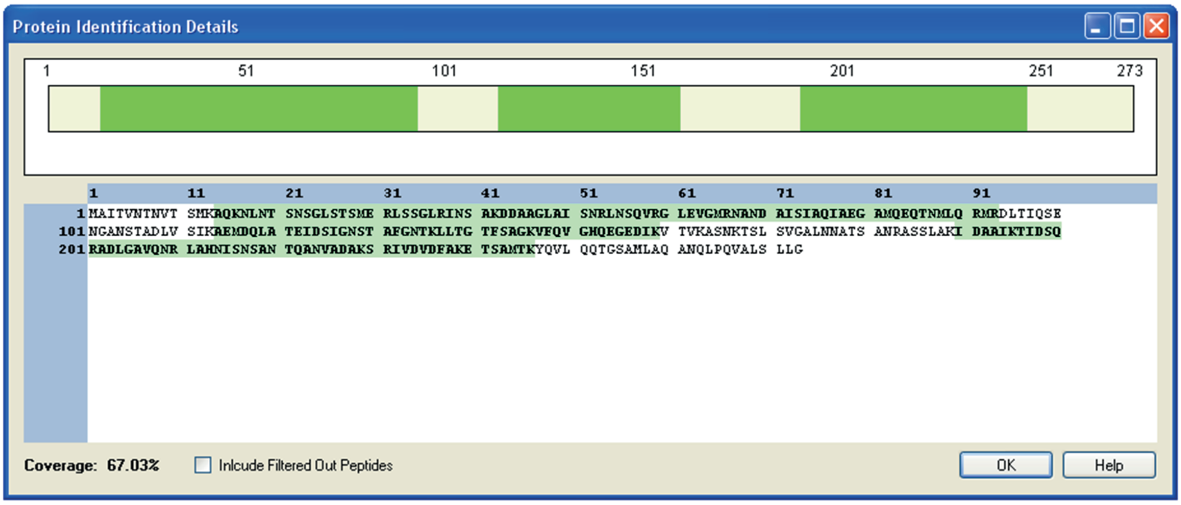

FlaB

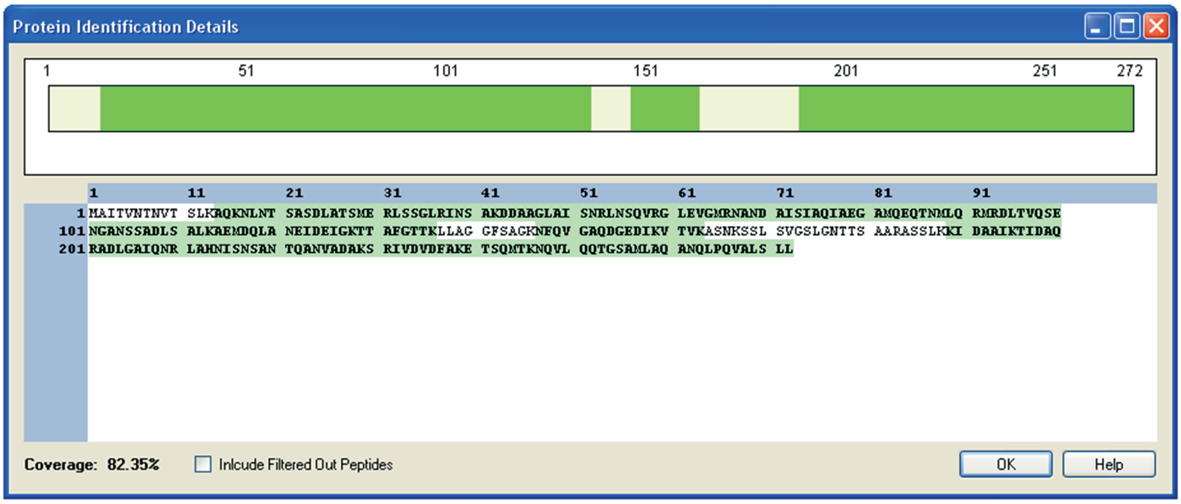

Supplemental Figure 2: Protein identification in *S. oneidensis* flagellar extract by mass spectrometry. The flagella extract of *S. oneidensis* was reduced, carboxyamidomethylated and digested with trypsin, and profiled by various mass spectrometry techniques. The figure represents the data from LTQ-orbitrap-XL (LC-NSI-CID-MS/MS). The resulting data was analyzed by Proteome Discoverer (Version 1.1, Thermo Scientific) using the Sequest algorism, and the detected peptides were searched against *S. oneidensis* flagella sequences SO_3237 (FlaB) and SO_3238 (FlaA). The peptides identified by the software are highlighted in green. 82% of the peptides from SO_3237 (FlaB) and 67% of the peptides from SO_3238 (FlaA) were identified from the protein extract.
