# Supplementary material for: Analyzing the Modification of the Shewanella oneidensis MR-1 Flagellar Filament
Source: PLoS One. 2013 Sep 6;8(9):e73444. doi: 10.1371/journal.pone.0073444 (PMC3765264; doi:10.1371/journal.pone.0073444)
Supplement: Figure S3 — HCD-MS/MS spectrum from L137LAGGFS@AGK146 plus a 522 Da modification. (@represents the site of modification). The HCD-MS/MS spectrum showed accurate mass of glycan-related ions correspond to dehydrated ions of the 522 Da modification, and oxonium ion from the 274 Da moiety of the 522 Da modification (A). The series of fragments from the 274 moiety were observed in the mass range between 100-300 m/z (B). An inset in the spectrum C shows a proposed structure of the 274 Da moiety (observed m/z of 274 Da moiety as an oxonium ion is m/z 275). Pentagon cartoon and grayed circle cartoons in the figure represents 274 Da- and 250 modifications, respectively. (PDF) [file pone.0073444.s003.pdf]

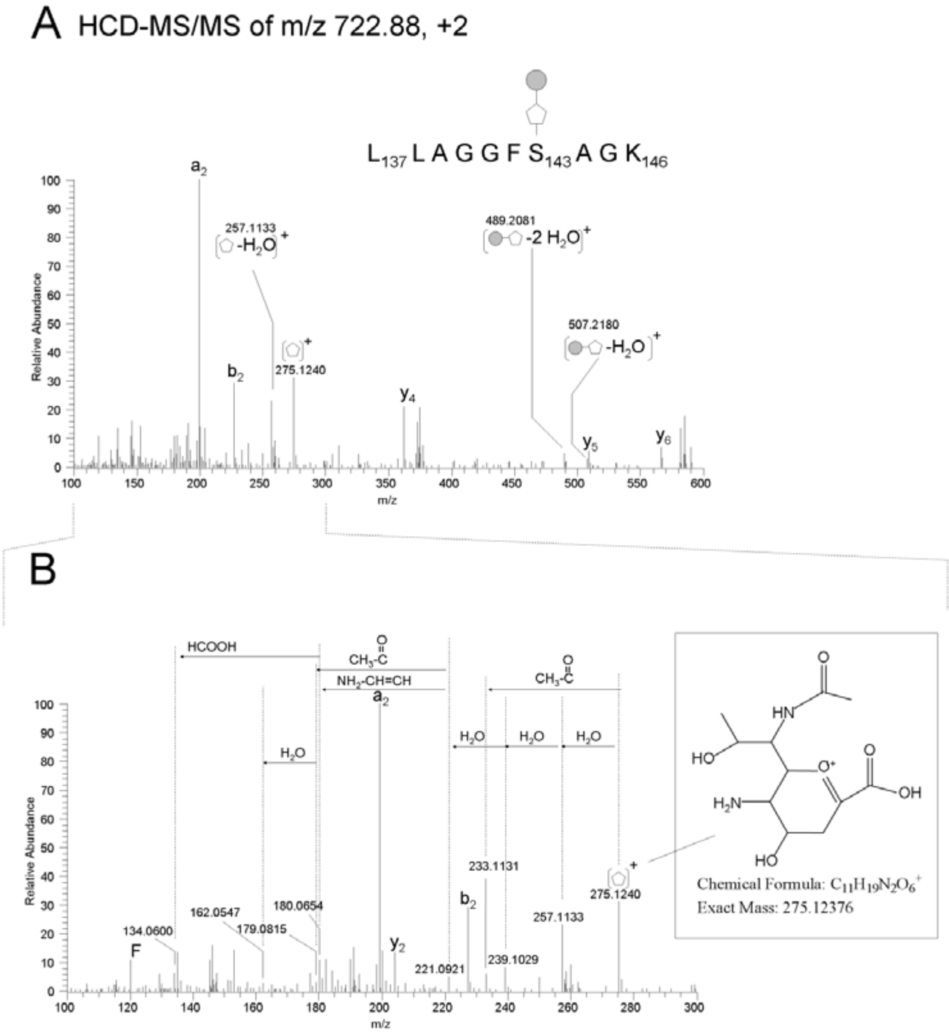

Supplemental Figure 3. HCD-MS/MS spectrum from L_{137}LTGTFS@AGK$_{146}$ plus a 522 Da modification. (@represents the site of modification). The HCD-MS/MS spectrum showed the accurate mass of glycan-related ions corresponding to dehydrated ions of the 522 Da modification, and an oxonium ion from the 274 Da moiety of the 522 Da modification (A). The series of fragments from the 274 moiety were observed in the mass range between 100-300 m/z (B). An inset in the spectrum B displays a proposed structure of the 274 Da moiety (observed m/z of 274 Da moiety as an oxonium ion is m/z 275). The pentagon cartoon and grayed circle cartoons in the figure represent the 274 Da- and 250 modifications, respectively.
