# Supplementary material for: Analyzing the Modification of the Shewanella oneidensis MR-1 Flagellar Filament
Source: PLoS One. 2013 Sep 6;8(9):e73444. doi: 10.1371/journal.pone.0073444 (PMC3765264; doi:10.1371/journal.pone.0073444)
Supplement: Figure S4 — HCD-MS/MS spectrum from L137LTGTFS@AGK146 plus a 510 Da modification (@represents the site of modification). The HCD-MS/MS spectrum showed accurate mass of glycan-related ions correspond to dehydrated ions of the 510 Da modification, and oxonium ion from the 274 Da moiety of the 510 Da modification (A). Figure 3 B represents the zoomed HCD spectrum between m/z 100 to 300. It should be noted that the signals observed from the peptide with 510 Da were very low compared to those with 524 Da or 538 Da modifications, dues to low abundance of the 510-Da modification occurs in the flagella. However, we detected main fragment ions from 274-Da modification. An inset in the spectrum C shows a proposed structure of the 274-Da moiety (observed m/z of 274 Da moiety as an oxonium ion is m/z 275). Pentagon cartoon and black-colored circle cartoons in the figure represent 274 Da- and 236 modifications, respectively. (PDF) [file pone.0073444.s004.pdf]

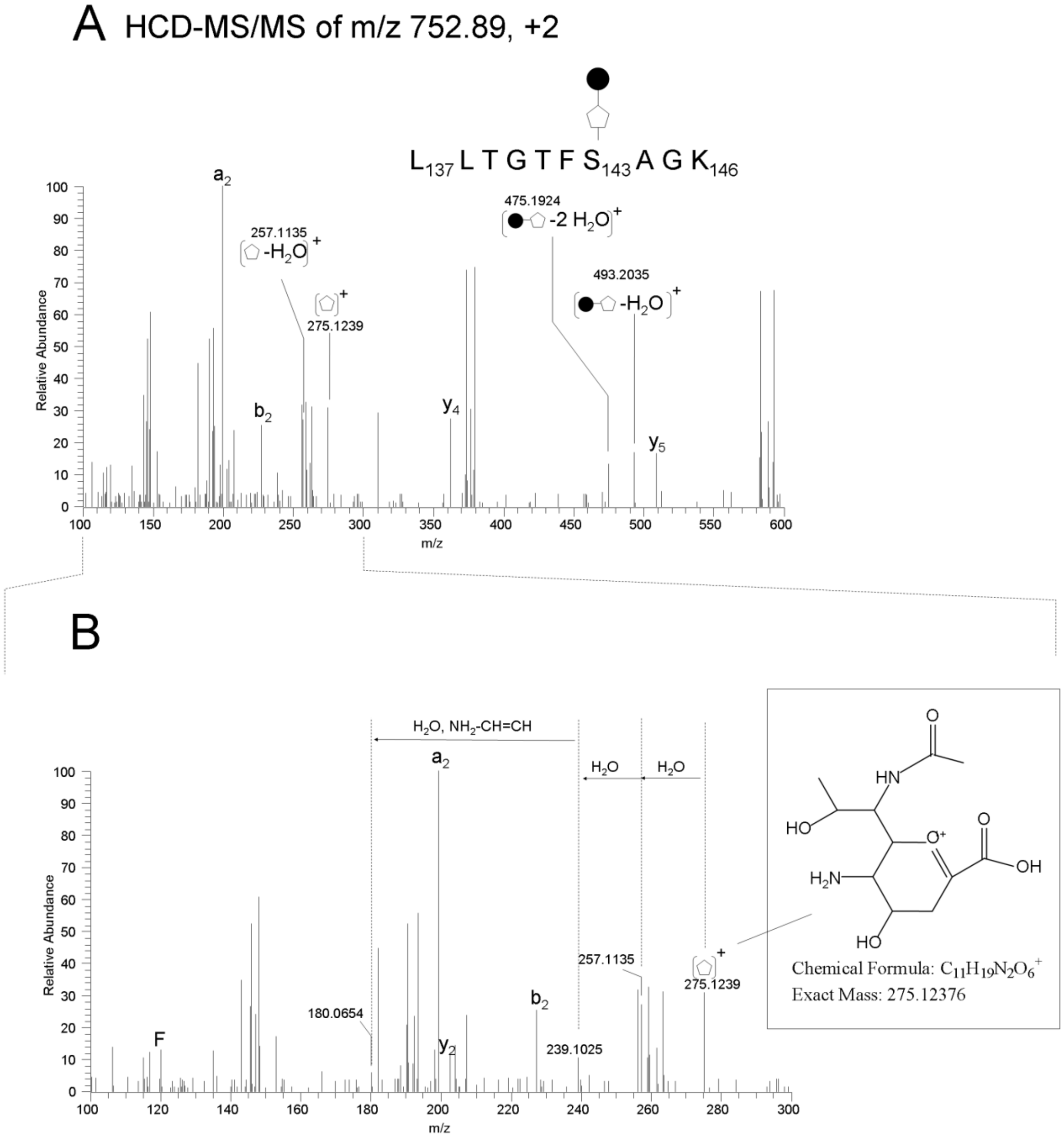

Supplemental Figure 4: HCD-MS/MS spectrum from L_{137}LTGTFS@AGK_{146} plus a 510 Da modification (@represents the site of modification). The HCD-MS/MS spectrum showed the accurate mass of glycan-related ions corresponding to dehydrated ions of the 510 Da modification, and an oxonium ion from the 274 Da moiety of the 510 Da modification (A). Figure 3 B represents the zoomed HCD spectrum between m/z 100 to 300. It should be noted that the signals observed from the peptide with 510 Da were very low compared to those with 524 Da or 538 Da modifications, due to low abundance of the 510-Da modification occuring in the flagellin. However, we detected main fragment ions from 274-Da modification. An inset in the spectrum B shows a proposed structure of the 274-Da moiety (observed m/z of 274 Da moiety as an oxonium ion is m/z 275). The pentagon cartoon and black-colored circle cartoons in the figure represent the 274 Da- and 236 modifications, respectively.
