# Supplementary material for: Analyzing the Modification of the Shewanella oneidensis MR-1 Flagellar Filament
Source: PLoS One. 2013 Sep 6;8(9):e73444. doi: 10.1371/journal.pone.0073444 (PMC3765264; doi:10.1371/journal.pone.0073444)
Supplement: Figure S5 — Glycopeptide purification by IT-TOF. The tryptic digest of S. oneidensis flagellar protein was prepared in larger scale and tryptic peptides/glycopeptides were purified from the sample by IT-TOF for further structural characterization of the modification by NMR. The sample elution was monitored on-line by IT-TOF mass spectrometry. The figure represents the spectra of the fraction 15, containing mostly L137LAGGFSAGK146 with the 538-Da modification, which was analyzed by NMR. (PDF) [file pone.0073444.s005.pdf]

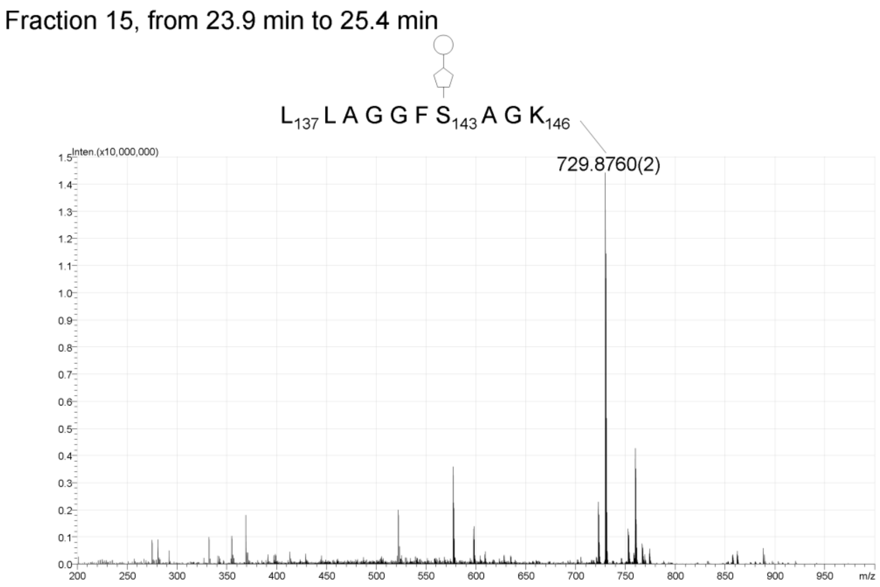

Supplemental Figure 5: Glycopeptide purification by IT-TOF. The tryptic digest of *S. oneidensis* flagellar protein was prepared in larger scale and tryptic peptides/glycopeptides were purified from the sample by IT-TOF for further structural characterization of the modification by NMR. The sample elution was monitored on-line by IT-TOF mass spectrometry. The figure represents the spectra of the fraction 15, containing mostly $L_{137}LAGGFSAGK_{146}$ with the 538-Da modification, which was analyzed by NMR.
