# Supplementary material for: Analyzing the Modification of the Shewanella oneidensis MR-1 Flagellar Filament
Source: PLoS One. 2013 Sep 6;8(9):e73444. doi: 10.1371/journal.pone.0073444 (PMC3765264; doi:10.1371/journal.pone.0073444)
Supplement: Figure S6 — 2D-NMR analysis of a glycopeptide fraction purified from S.oneidensis. Two-Dimensional gradient-enhanced COSY NMR spectrum of Fraction 15, containing mostly L137LAGGFSAGK146 with the 538-Da modification, run at 800 MHz and 25 °C. The dotted lines connect the two H-3 protons and the H-4 proton of the nonulosonic acid detected in MS. (PDF) [file pone.0073444.s006.pdf]

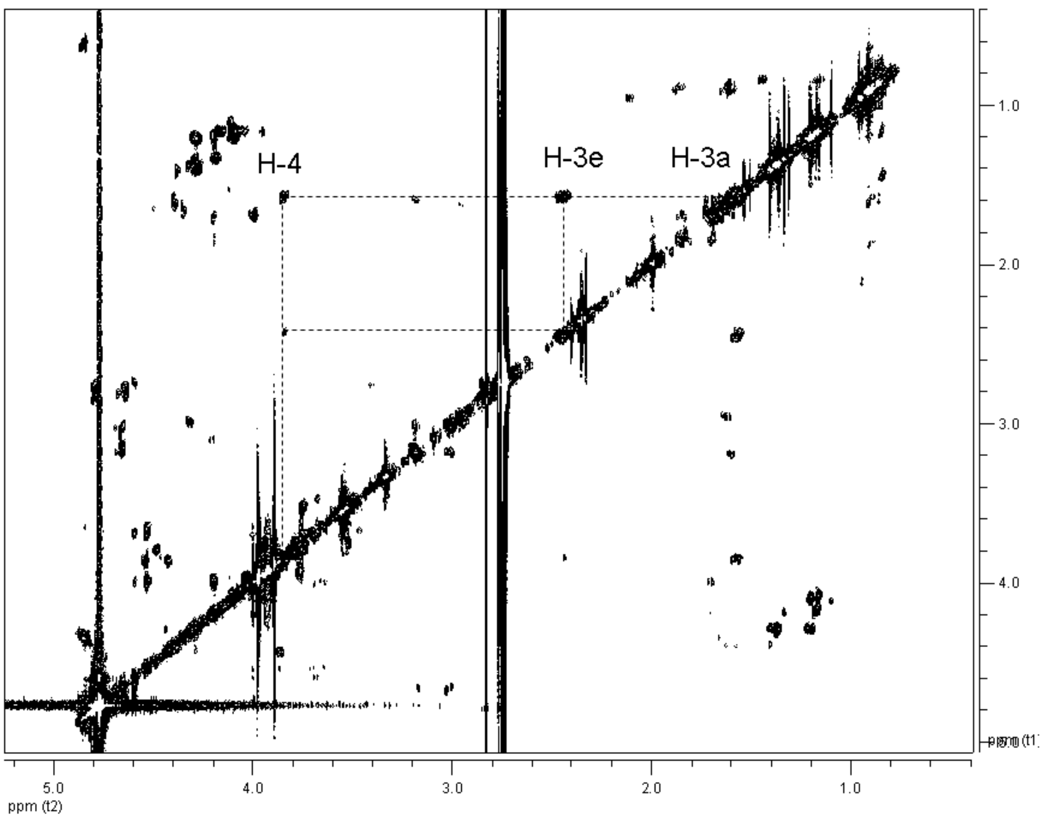

Supplemental Figure 6: 2D-NMR analysis of a glycopeptide fraction purified from *S.oneidensis*. The image shows a two-dimensional gradient-enhanced COSY NMR spectrum of fraction 15, containing mostly $L_{137}LAGGFSAGK_{146}$ with the 538-Da modification, run at 800 MHz and 25 °C. The dotted lines connect the two H-3 protons and the H-4 proton of the nonulosonic acid detected in MS.
