# Supplementary material for: Analyzing the Modification of the Shewanella oneidensis MR-1 Flagellar Filament
Source: PLoS One. 2013 Sep 6;8(9):e73444. doi: 10.1371/journal.pone.0073444 (PMC3765264; doi:10.1371/journal.pone.0073444)
Supplement: Figure S7 — Alignment of the variable regions of Shewanella flagellins. Variable regions of S. oneidensis MR-1 FlaA and FlaB (aa 101-189) were aligned to corresponding flagellin sequences of Shewanella spp. harbouring flagellins with a similar domain structure (comprising between 260 and 280 aa). Modified residues in FlaB are marked with arrows and in red. At some positions, several flagellins have a threonine instead of a serine residue (marked in green) that might be similarly modified. Invariable residues are highlighted in yellow, and positions with a single amino acid variation are marked in grey. Locus tag coding: Swoo, S. woodyi; Ssed, S. sediminis; Shew, S. loihica; Shal, S. halifaxensis; Sden, S. denitrificans; Sputw3181, S. sp. W3-18-1; Shewana3, S. sp. ANA-3; Sbal183, S. baltica OS183; Shew185, S. baltica OS185; Sput200, S. putrefaciens 200. (PDF) [file pone.0073444.s007.pdf]

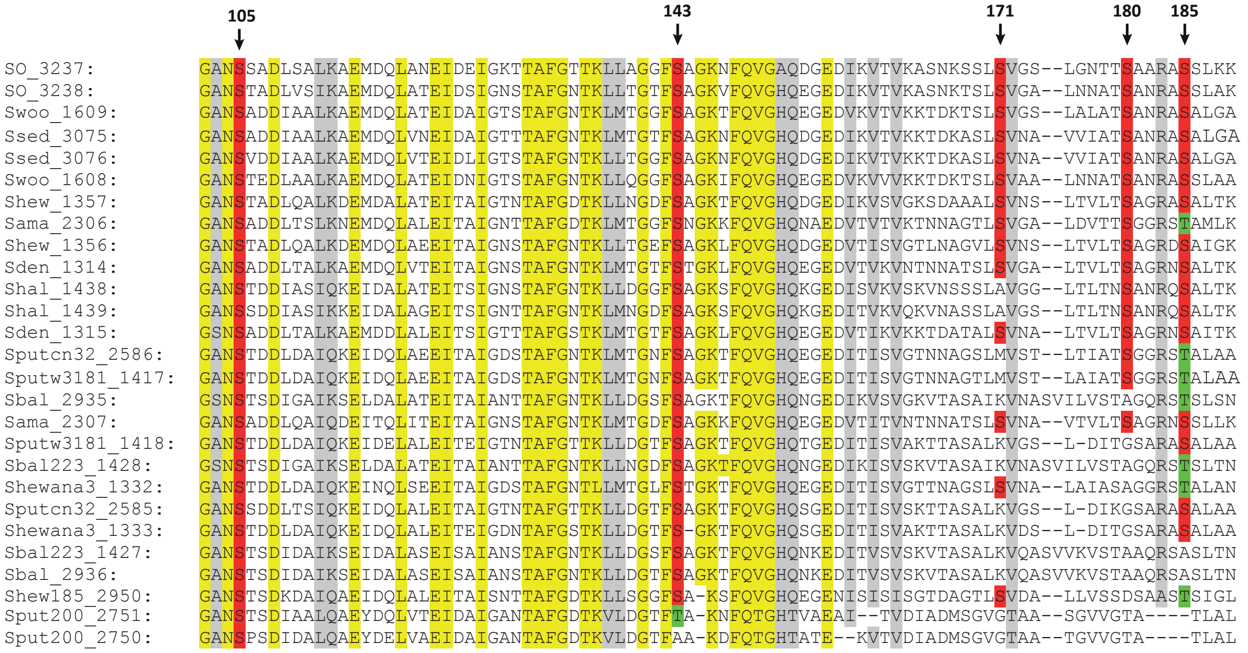

Supplemental Figure 7: Alignment of the variable regions of *Shewanella* flagellins. Variable regions of *S. oneidensis* MR-1 FlaA and FlaB (aa 101-189) were aligned to corresponding flagellin sequences of *Shewanella* spp. harbouring flagellins with a similar domain structure (comprising between 260 and 280 aa). Modified residues in FlaB are marked with arrows and in red. At some positions, several flagellins have a threonine instead of a serine residue (marked in green) that might be similarly modified. Invariable residues are highlighted in yellow, and positions with a single amino acid variation are marked in grey. Locus tag coding: Swoo, *S. woodyi;* Ssed, *S. sediminis*; Shew, *S. loihica*; Shal, *S. halifaxensis*; Sden, *S. denitrificans*; Sputcn32, *S. putrefaciens* CN-32; Sputw3181, *S.* sp. W3-18-1; Shewana3, *S.* sp. ANA-3; Sbal183, *S. baltica* OS183; Shew185, *S. baltica* OS185; Sput200, *S. putrefaciens* 200.
